# Supplementary material for: Are deprivation-specific cancer survival patterns similar according to individual-based and area-based measures? A cohort study of patients diagnosed with five malignancies in England and Wales, 2008–2016
Source: BMJ Open. 2022 Jun 9;12(6):e058411. doi: 10.1136/bmjopen-2021-058411 (PMC9189835; doi:10.1136/bmjopen-2021-058411)
Supplement: Author's manuscript [file bmjopen-2021-058411.draft_revisions.pdf]

BMJ Open is committed to open peer review. As part of this commitment we make the peer review history of every article we publish publicly available.

When an article is published we post the peer reviewers' comments and the authors' responses online. We also post the versions of the paper that were used during peer review. These are the versions that the peer review comments apply to.

The versions of the paper that follow are the versions that were submitted during the peer review process. They are not the versions of record or the final published versions. They should not be cited or distributed as the published version of this manuscript.

BMJ Open is an open access journal and the full, final, typeset and author-corrected version of record of the manuscript is available on our site with no access controls, subscription charges or pay-per-view fees (http://bmjopen.bmj.com).

If you have any questions on BMJ Open's open peer review process please email info.bmjopen@bmj.com

BMJ Open

Are deprivation-specific cancer survival patterns similar according to individual- and area-based measures? A cohort study of patients diagnosed with five malignancies in England & Wales, 2008-2016

Journal:	*BMJ Open*
Manuscript ID	bmjopen-2021-058411
Article Type:	Original research
Date Submitted by the Author:	15-Oct-2021
Complete List of Authors:	Woods, Laura; London School of Hygiene & Tropical Medicine, Department of Non-Communicable Disease Epidemiology<br>Belot, Aurelien; London School of Hygiene & Tropical Medicine<br>Atherton, Iain; Napier University SAFSL<br>Ellis-Brookes, Lucy; Public Health England<br>Baker, Matthew ; National Cancer Research Institute, Consumer Forum<br>Ingleby, Fiona; London School of Hygiene & Tropical Medicine, Department for Non-Communicable Disease Epidemiology
Keywords:	Epidemiology < ONCOLOGY, Adult oncology < ONCOLOGY, PUBLIC HEALTH, QUALITATIVE RESEARCH

SCHOLARONE™
Manuscripts

I, the Submitting Author has the right to grant and does grant on behalf of all authors of the Work (as defined in the below author licence), an exclusive licence and/or a non-exclusive licence for contributions from authors who are: i) UK Crown employees; ii) where BMJ has agreed a CC-BY licence shall apply, and/or iii) in accordance with the terms applicable for US Federal Government officers or employees acting as part of their official duties; on a worldwide, perpetual, irrevocable, royalty-free basis to BMJ Publishing Group Ltd ("BMJ") its licensees and where the relevant Journal is co-owned by BMJ to the co-owners of the Journal, to publish the Work in this journal and any other BMJ products and to exploit all rights, as set out in our licence.

The Submitting Author accepts and understands that any supply made under these terms is made by BMJ to the Submitting Author unless you are acting as an employee on behalf of your employer or a postgraduate student of an affiliated institution which is paying any applicable article publishing charge ("APC") for Open Access articles. Where the Submitting Author wishes to make the Work available on an Open Access basis (and intends to pay the relevant APC), the terms of reuse of such Open Access shall be governed by a Creative Commons licence – details of these licences and which Creative Commons licence will apply to this Work are set out in our licence referred to above.

Other than as permitted in any relevant BMJ Author's Self Archiving Policies, I confirm this Work has not been accepted for publication elsewhere, is not being considered for publication elsewhere and does not duplicate material already published. I confirm all authors consent to publication of this Work and authorise the granting of this licence.

For peer review only

Research Paper

Are deprivation-specific cancer survival patterns similar according to individual- and area-based measures? A cohort study of patients diagnosed with five malignancies in England & Wales, 2008-2016

Laura M Woods*[1], Aurélien Belot[1], Iain Atherton[2], Lucy Ellis-Brookes[3], Mat Baker[4], Fiona C Ingleby[1]

1. Inequalities in Cancer Outcomes Network, Department of Non-Communicable Disease Epidemiology, Faculty of Epidemiology and Population Health, London School of Hygiene and Tropical Medicine, London, UK

2. School of Health & Social Care, Edinburgh Napier University, Edinburgh, UK

3. National Cancer Registration and Analysis Service, Public Health England, London, UK

4. National Cancer Research Institute Consumer Forum, London, UK

* Corresponding author:

laura.woods@lshtm.ac.uk

ORCID https://orcid.org/0000-0002-2178-1577

Twitter handle: @icon_lshtm

For peer review only

Abstract

Objective: To investigate the relative influence of individual- (*person*) vs. area-based (*place*) measures of deprivation upon cancer survival differentials for three separate socio-economic dimensions: income, education and occupation.

Design: Cohort study

Setting: Data from the Office for National Statistics (ONS) Longitudinal Study of England and Wales, UK, linked to the National Cancer Registration Database

Participants: Patients diagnosed with cancers of the colorectum, breast, prostate, bladder or with Non-Hodgkin Lymphoma (NHL) during the period 2008-2016

Primary and secondary outcome measures: Differentials in net survival between groups defined by individual wage, occupation and education compared to those obtained from corresponding area-level metrics using the English and Welsh Indices of Multiple Deprivation (IMD).

Results: Survival was negatively associated with area-based deprivation irrespective of the type analysed, although a trend from least to most deprived was not always observed. There was an absence of a 'gradient' in survival according to individually-measured socio-economic groups. The magnitude of differentials was similar for area-based and individually-derived measures of deprivation, which was unexpected.

Conclusion: These unique data suggest that the socio-economic influence of *person* is different to that of *place* with respect to cancer outcomes. This has implications for health policy aimed at reducing inequalities. Further research should address the existence of contextual effects using a modelling approach as well as define the particular characteristics of areas with poor outcomes in order to inform policy intervention.

For peer review only

Strengths and limitations of this study

- We analysed a unique, representative, cohort of England and Wales within which it was possible to classify individuals by both their area-based deprivation score and individual socioeconomic group.
- We used newly derived, individual life tables to estimate underlying mortality for each individual socioeconomic group.
- We used a generic life table for area-based deprivation analyses because education- and occupation-specific life tables were unavailable, but also to prioritise the use of mortality rates derived from the ONS-LS itself.
- We estimated individual income on the basis of recorded occupation due to the absence of directly measured data on earnings.
- Our study design enabled us to assess the relative impact of person vs. place upon socio-economic differentials in cancer outcomes.

For peer review only

Introduction

It has been widely documented that there are long-standing, persistent inequalities in cancer outcomes between individuals living in more deprived areas and those living in less deprived areas in higher income countries.[1-7] These inequalities may partly explain why cancer survival in the UK is lower than other similar settings, where socio-economic differentials tend to either be smaller or explicable by factors such as stage of disease at presentation.[8-10] The public health impact of these disparities is considerable as shown by the large number of associated avoidable deaths[11] and the influence this body of work has had on UK health policy over a period of more than twenty years.[12-15]

Despite their widespread use, the exact meaning of differentials observed between geographic areas with contrasting levels of deprivation have been variably interpreted. Most often, poorer outcomes amongst *'persons living in deprived areas'* has been treated as a proxy for poorer outcomes amongst *'deprived persons'*[16] without consideration that area-level deprivation could have a separate and independent influence over and above an individual's own personal characteristics. This has led in turn to an implicit assumption that the inequalities observed between affluent and deprived areas are most likely diluted versions of the 'real' (unknown) differences between individuals of different socio-economic groups, perhaps driven by the fact that, and consistent with the ecological fallacy, larger differentials are observed when the size of the geographical unit of analysis is smaller.[17, 18]

Observed trends at the area-level combined with an inherent assumption of a dilution effect has thus tended to steer policy-related research into the individual domain, for example raising individual symptom awareness in these populations,[19] increasing the probability of early stage diagnosis through screening, and ensuring appropriate and effective treatment is given to patients living in more deprived localities.[19, 20] Studies consistently documenting poorer outcomes in more deprived areas has also fuelled change in the way funding is allocated, causing them to be tied to specific assessments of unmet need, along with measurable, mostly individually-orientated, goals and mechanisms by which health inequalities might be narrowed.[12]

Only a relatively small body of research has examined cancer outcomes using individual, personal, measures of socio-economic status,[21] principally because data sources suitable for such an analysis are fewer and, or, more difficult to access.[22] Socio-economic differentials have relatively infrequently been considered as geographical phenomenon driven by locality-based factors such as travel time to hospital, access to GP services, except in especially rural settings outside the UK.[23] Similarly, the influence of community characteristics including social capital or social cohesion has not been widely considered. These social environmental influences on health outcomes, if important, are likely to be driven by a separate set of factors to those acting purely at an individual level.

For peer review only

Added to this, throughout the literature on inequalities there has tended to be a singular focus on a single dimension of deprivation, normally income, or a single composite score.[22] Relatively few studies have considered whether the different dimensions of deprivation have a similar or different effect, implicitly assuming that a single measure is sufficient to examine the underlying phenomenon of interest. A broader consideration of the relative contributions of wealth, status and power[24] upon cancer outcomes could help to clarify the mechanisms by which inequalities arise and are perpetuated.

Using the Office for National Statistics Longitudinal Study (ONS-LS) we have shown that the concordance between individual socio-economic group and the deprivation present in the small area of residence is relatively low amongst cancer patients for three separate domains, but most especially for income.[25] Interpreting area-based analyses derived from a single measure as broadly representative of individual inequalities therefore risks overlooking some important sub-groups of individuals. The objective of this follow-up study was to compare inequalities according to individual and area-based measures, contrasting the impact of income, occupation and education upon survival.

For peer review only

Methods

Cancer patient cohort

We analysed records from the national cancer registry[26] individually linked to the Office for National Statistics Longitudinal Study (LS).[27] The LS sample is a random sample clustered by date of birth.[25, 28] Census data for cohort members are available from the 1971 census through to the 2011 census. The ONS LS also links life events data, including cancer registrations and deaths of members. The analysis cohort for this study included LS members present at either or both 2001 and 2011 census, and diagnosed with a first primary malignant cancer diagnosis between 1 January 2008 and 30 April 2016 at ages 20-100 years old. We examined five common cancer types: breast (ICD-10 code C50), prostate (C61), colorectal (C18-21), Non-Hodgkin Lymphoma (C82-86), and bladder (C67). These specific malignancies were selected as it has been demonstrated that they exhibit significant area-based socio-economic differentials for both sexes.[5] A small number (<20) of sex-site errors, and also a small number (<30) of men with breast cancer were excluded.

Area-level deprivation

The Indices of Multiple Deprivation for England[29] and Wales[30] were used to measure area-based deprivation. We used the income, employment (i.e., occupation) and education domains for the Lower Super Output Area of residence, using the temporally closest score to each census. For the 2001 census, this was the English IMD2004 and the Welsh metrics reported in 2005. For the 2011 census, this was the English IMD2015 and the Welsh metrics reported in 2014. Each index was linked to the data as quintiles of the national distribution

of areas, and three deprivation groups were created for the purposes of this analysis: least deprived (quintiles 1 & 2), mid (quintile 3) and most deprived (quintiles 4 & 5).

Individual-level socio-economic variables

Individual data on age, sex, qualifications and occupation were extracted directly from census data for each patient. Occupation type was derived using the 3-group version of the National Statistics Socio-Economic Classification (NS-SEC) to ensure sufficient numbers to enable statistical analysis. These are technical, routine and manual occupations; intermediate occupations; or higher managerial, administrative and professional occupations.[29]

Education level was categorised as one of three groups based on standard levels of English and Welsh qualifications used in the census: no qualifications; school or college qualifications (GCSEs, A-levels, apprenticeships, vocational qualifications or equivalent) and degree qualifications (degree-level education or higher).

Weekly income (GBP) was estimated for each individual following the method formulated by Clemens and Dibben.[31] We took a data-driven approach to adjust income for those aged over 60, who were most likely to be retired. We adjusted these income estimates using the observed annualised percentage decreases in income for those aged over 60 reported by the English Longitudinal Study of Ageing.[32] After applying this correction, LS members were categorised into three groups by estimated income: lowest income (quintiles 1 & 2), middle income (quintile 3) and highest income (quintiles 4 & 5). Quintiles were calculated based on all available LS members (i.e., not just cancer patients), separately for each sex.

Data were not available from the 2011 census for a small proportion of individuals; mostly accounted for by those who were diagnosed with cancer between 2008-2011 and died prior to the 2011 census.[25] Where possible, data from the 2001 census were used for these individuals. Missing data on qualifications or occupation (which includes long-term unemployed and students for the 3-group version of the NS-SEC as recommended by the NS-SEC guidelines[29]), were completed where possible by proxy, using another adult resident in the household (usually household head). Following this procedure, 6%, <1%, and 5% of records were missing individual deprivation data for occupation, education, and income respectively. These individuals were excluded.

Survival analysis

Analyses were carried out separately for men and women. We analysed survival time (days between date of diagnosis and date of death or censoring) as a function of patient age and either socio-economic group or area-based deprivation group, adjusted for the ‘expected’ mortality. Data were censored on the 31-Dec-2017, the date of the most recent linkage of the ONS-LS to mortality records.

For peer review only

We report net survival and 95% confidence intervals, calculated using the non-parametric Pohar-Perme estimator[33] with the 'relsurv'[34] package in R v.3.6.3.[35] This is the most widely used, consistent estimator of net survival. Net survival is the survival probability patients would experience if their only possible cause of death were cancer. Net survival estimates are independent of underlying other-cause mortality and thus reflect cancer-specific prognosis. We account for underlying deaths from other causes using 'expected' mortality estimates for each individual socio-economic group, which we extracted from life tables that we derived from this same ONS-LS cohort.[36] Expected mortality for the area-level deprivation analyses used life tables based on the overall ONS-LS cohort.

Net survival is reported as age-standardised estimates (Age-Standardised Net Survival, ASNS), derived using ICSS weights for age groups, with the youngest two groups merged together (i.e. 15-54, 55-64, 65-74, 75+) to allow for the lower numbers in the youngest age groups in this population sample. For each deprivation measure and each cancer type, we calculated the arithmetic difference in survival between the most affluent and most deprived groups as the 'survival gap' (irrespective of which group displayed the highest or lowest survival).

Patient and Public Involvement

This study was first presented to patient representatives at the NCRI Consumer Forum 'Dragon's Den' in 2017, where Mr Matthew Baker, along with nine other members of the public, provided input and ideas for the approach and methodology. Mr Baker has acted as Non-Academic Co-Investigator, helped to develop the study protocol and implement the research plans. He has attended all project meetings to provide insights on decision making as the project progressed. Following the production of results the whole project team have worked on the dissemination and discussion of the results at both a further Dragon's Den meeting, and in online forums with policy makers. The research will also been presented online to members of the public who engaged with the topic via a specifically-targeted Facebook marketing campaign.

Results

Overall, 5,551 men and 5,284 women were included in the analyses. The cohort was broadly representative of the population from which it was drawn: the sex-specific age distribution of cases for each cancer site are similar to that of the overall population of England and Wales (Table 1). The data included a sufficiently large number of deaths by cancer and sex to enable net survival estimation (Table 2). Similar proportions of men and women died within 1 and 5 years of diagnosis in the ONS-LS and in England and Wales.

Socio-economic variations in net survival were observed at both 1 and 5 years after diagnosis for both sexes and for each cancer site (Figures 1-3).

Survival tended to be negatively associated with area-level deprivation irrespective of the type analysed, with estimates in the most deprived areas between 0.5% and 12.9% lower than in the least deprived areas at 1

For peer review only

year since diagnosis, and between 1.9% and 17.7% lower at 5 years. The only exceptions were for women with NHL, where area-based survival was not associated with increasing deprivation, and for men with colorectal cancer across occupation at 1-year. Differences across area-based income measures tended to show the most consistent and strongest negative associations.

Patterns according to individual socio-economic group were more mixed. The association between survival and deprivation was generally weaker for occupation than for other types of socio-economic variable amongst men and, to a lesser extent, women. Percentage point differences in ASNS 1 year after diagnosis between individuals with degree-level qualifications and no qualifications ranged from 2.3% to 15.9%, amongst those with the highest and lowest incomes from -2.5% to 17.2%, and from -0.1% to 12.5% between those working in manual compared to professional occupations.

Differentials between individual-level socio-economic groups in comparison to area-based deprivation quintiles are plotted against one another as the 'survival gap' in Figure 4. The diagonal line indicates an equal extent of survival inequality measured in individual-level and area-level analysis.

For men with colorectal and prostate cancer, the deprivation 'gap' was of a similar or slightly smaller magnitude between individual socio-economic groups compared to area-based quintiles, for both 1-and 5-year survival, for education, occupation and, to a lesser extent, income. Colorectal cancer differentials amongst women were greater using individual-based measures than area-based measures 1 year after diagnosis, but more similar 5 years after diagnosis, for all three types of deprivation. Breast cancer inequalities were of a similar magnitude 1 year after diagnosis for all types of deprivation, but larger for area-based measures after 5 years in comparison to those observed between individual socio-economic groups. The deprivation gap tended to be smallest overall for men with prostate cancer and, to a lesser extent, women with breast cancer.

Bladder and NHL are lower incidence malignancies so the number of cases and deaths we examined were much smaller. As such, the survival estimates for these cancers have wider variance and the interpretation of these data should be treated with caution. Amongst men patterns for NHL were similar to the more common cancer sites. Amongst women with NHL an unexpected reverse trend was seen between area-based educational deprivation and survival, where more deprived women had better outcomes. More deprived bladder cancer patients displayed poorer outcomes amongst both men and women. There was a suggestion that area-based measures had a greater impact compared to individual socio-economic group for men with bladder cancer, but patterns for women were similar between area-based and individual measures.

Discussion

We have quantified non-parametric net survival for five cancers previously shown to have substantial area-level deprivation gaps in survival,[5] comparing inequalities derived using area-based deprivation measures to those obtained using individual measures of SES. Consistent with the literature, survival was most often lower

For peer review only

amongst those from more deprived localities irrespective of the type of deprivation analysed.[6, 17] By contrast, there was an unexpected lack of overall trend of lower survival across the spectrum of individual socio-economic groups as well as a notable lack of trend between individual income groups. Our results suggest that the role of person versus place differs with respect to cancer outcomes and that the underlying reasons for this warrant further investigation.

Individual vs. area-based differentials

We calculated deprivation gaps in cancer survival in order to evaluate whether differentials between deprived and affluent individuals were larger, smaller, or similar to those between deprived and affluent populations. The similarity of the magnitude of the deprivation gaps across area- and individual-based measures suggests no evidence for a dilution effect, which was unexpected. Rather, these data are more supportive of the existence of two separate effects for cancer outcomes, one of person (individual effect), another of place (area-based effect). Our results are consistent with our previous findings which showed that deprived persons frequently resided in non-deprived areas,[25] and speaks against interpretations of area-based data where poorer health outcomes amongst deprived populations have been assumed to arise simply from poorer outcomes amongst deprived persons (dilution effect). The exception to this pattern is women's 1-year survival, where there is some suggestion of dilution for bladder and colorectal cancer, although these data points had wide confidence intervals. The smaller differentials we observed for prostate and breast cancers are likely in part to constitute a form of ceiling effect, since differentials tend to be smaller when survival is high, even if the excess hazard ratio is of a similar magnitude to other cancers with lower survival.

Domains of deprivation

For the most part the different measures of deprivation (income, education, occupation) exerted a broadly similar effect on cancer outcomes in area-based analyses. This has been previously observed[17] and is also consistent with sociological theory which states that socio-economic status arises from three inter-related domains: class (broadly reflected by education), state (occupation) and power (income).[24] At the individual level, cancer outcomes were correlated with individual occupation and education for both men and women. Higher individual income amongst men was counter-intuitively associated with poorer outcomes in some analyses, and in others displayed no discernible trend. These results were somewhat unexpected, especially given the clear association observed between area-based income deprivation and cancer outcomes in these same patients. These observations could be explained in part by the imputation of individual income from occupational codes. Alternatively, it is possible that this is a threshold effect, where variations in income above a certain level are not strongly associated with cancer outcomes. This suggests that the existing literature on income deprivation patterns may be picking out differentials between populations with differing proportions of persons on very low incomes.

Strengths and limitations

For peer review only

Our study has a number of strengths. First, we used a unique, representative cohort of England and Wales within which we were able to classify individuals by both area-based deprivation score and individual socioeconomic group, so as to assess area- and individual-level patterns within the same cohort of patients. We used newly derived individual life tables[36] to estimate underlying mortality for each individual socioeconomic group, matched to the most up-to-date methodology for estimating non-parametric survival from cancer.[33] For prostate, breast and colorectal cancers we were able to obtain sample sizes sufficiently large to confidently compare outcomes and the ‘survival gaps’ between the two different approaches. Numbers for bladder cancer and non-Hodgkin lymphoma were smaller (smallest group 202 cases with approximately 80 deaths). Patterns for bladder cancer lent weight to our overall conclusions, whilst those for NHL were less consistent. Limitations to our approach include the use of a generic life table for the area-based analyses, as well as the need to estimate individual income on the basis of recorded occupation. Although life tables for England and Wales as a whole derived from quintiles of area-based income deprivation are available,[37] these have not been derived specifically for education and occupation sub-domains. Further, our methods prioritised using life tables derived from the same cohort, so as not to introduce a bias from the use of a national life table: whilst the LS is representative of the overall population it is still only a small sample of the whole of England and Wales combined, and so it was more appropriate to extract observed rates of death from the cohort itself. The income variable for individuals was necessarily an estimate, since this information is not directly collected in the UK Census. However, we used an externally-validated method,[31] which was based on a separate measure of occupation[29] to the employment domain, as well as age and sex, in order to generate the most accurate estimate as possible.

Policy implications & further research

Our results have significance for public health policy on inequalities, demonstrating that there is unlikely to be a simple correspondence between reducing differentials between more and less-deprived areas and improving outcomes for individually-deprived persons. Further research is required to establish the mechanisms by which these patterns arise. Whilst area-based measures are exactly that, based upon areas rather than persons, they are still derived from observed proportions of individual people experiencing or having specific personal characteristics of low socio-economic status (for example, income benefits, unemployment status, or lack of formal qualifications). As such, they do not include any environmental measures of deprivation such as access to services, travel time, travel costs, number of GPs or specialist oncologists per capita. Furthermore, they do not consider the social or community setting, in that they do not and cannot measure factors such as social capital or social cohesion. More detailed analyses are therefore required to better understand area-based patterns: first to establish using multivariable approaches if there is a specific contextual effect of place above and beyond the influence of the socioeconomic status of a person, and second, to discover the particular intrinsic characteristics of places (rather than just the people residing within them) where survival outcomes are poor.

For peer review only - http://bmjopen.bmj.com/site/about/guidelines.xhtml

Conclusion

We have conducted a unique analysis of cancer survival with respect to individual and area-based measures of deprivation. These data suggest that the influence of person and place upon cancer outcomes warrants further investigation as part of a public health strategy to reduce cancer, as well as wider health, inequalities. Further investigation of both contextual effects and the particular characteristics of areas with poor outcomes would enable the derivation of more accurate hypotheses about the underlying causes of inequalities, and elucidate potential avenues for policy intervention.

For peer review only

Additional information

Acknowledgements

The permission of the Office for National Statistics to use the Longitudinal Study is gratefully acknowledged, as is the help provided by staff of the Centre for Longitudinal Study Information & User Support (CeLSIUS). CeLSIUS is supported by the ESRC Census of Population Programme under project ES/V003488/1. The authors alone are responsible for the interpretation of the data in this paper. This work uses data provided by patients and collected by the NHS as part of their care and support. Using patient data is vital to improve health and care for everyone. There is huge potential to make better use of information from people's patient records, to understand more about disease, develop new treatments, monitor safety, and plan NHS services. Patient data should be kept safe and secure, to protect everyone's privacy, and it's important that there are safeguards to make sure that it is stored and used responsibly. Everyone should be able to find out about how patient data is used.

Authors' contributions

LW, AB and IA conceived the study, developed the proposal, acquired the funding, ethical approval and data. They co-managed the conduct of the research and interpretation of the results. FI conducted all analyses, assisted with the interpretation of the data and drafted the initial manuscript. LW wrote the final version and led on the manuscript submission. LEB and MB assisted in the acquisition of funding, the interpretation of the data, and commented on drafts of the manuscript. All authors have approved the final version and agree to be accountable for all aspects of this work.

Ethics approval

Ethics approval for this study was obtained from the London School of Hygiene and Tropical Medicine Ethics Online Application 14600; approved 01/02/2018. Data presented for England and Wales and presented in Tables 1 and 2 were obtained following statutory approval from the Confidentiality Advisory Group (CAG) of the Health Research Authority (HRA): PIAG 1–05(c) 2007.

Data availability

Data are not publicly available but can be accessed via appropriate application to the ONS Longitudinal Study (https://www.ucl.ac.uk/epidemiology-health-care/research/epidemiology-and-public-health/research/health-and-social-surveys-research-group/studies-44). This work contains statistical data from ONS which is Crown Copyright. The use of the ONS statistical data in this work does not imply the endorsement of the ONS in relation to the interpretation or analysis of the statistical data. This work uses research datasets which may not exactly reproduce National Statistics aggregates. Data presented for England and Wales in Tables 1 and 2 are available via application to the Public Health England Office

For peer review only

for Data Release (https://www.gov.uk/government/publications/accessing-public-health-england-data/about-the-phe-odr-and-accessing-data).

Competing interests

The authors declare no competing interests.

Funding information

This work was supported by the Economic and Social Research Council (ES/S001808/1).

For peer review only

References

1. Coleman MP, Rachet B, Woods LM, Mitry E, Riga M, Cooper N, Quinn MJ, Brenner H, Esteve J. Trends and socioeconomic inequalities in cancer survival in England and Wales up to 2001. *Br J Cancer* 2004;**90**: 1367-73.

2. Finke I, Behrens G, Maier W, Schwettmann L, Pritzkuleit R, Holleczek B, Kajuter H, Gerken M, Mattutat J, Emrich K, Jansen L, Brenner H, German Cancer Survival Working G. Small-area analysis on socioeconomic inequalities in cancer survival for 25 cancer sites in Germany. *Int J Cancer* 2021.

3. Fowler H, Belot A, Njagi EN, Luque-Fernandez MA, Maringe C, Quaresma M, Kajiwara M, Rachet B. Persistent inequalities in 90-day colon cancer mortality: an English cohort study. *Br J Cancer* 2017;**117**: 1396-404.

4. Luque-Fernandez MA, Redondo-Sanchez D, Rodriguez-Barranco M, Chang-Chan YL, Salamanca-Fernandez E, Nunez O, Fernandez-Navarro P, Pollan M, Sanchez MJ. Socioeconomic inequalities in colorectal cancer survival in southern Spain: a multilevel population-based cohort study. *Clin Epidemiol* 2020;**12**: 797-806.

5. Rachet B, Ellis L, Maringe C, Chu T, Nur U, Quaresma M, Shah A, Walters S, Woods L, Forman D, Coleman MP. Socioeconomic inequalities in cancer survival in England after the NHS cancer plan. *Br J Cancer* 2010;**103**: 446-53.

6. Exarchakou A, Rachet B, Belot A, Maringe C, Coleman MP. Impact of national cancer policies on cancer survival trends and socioeconomic inequalities in England, 1996-2013: population based study. *BMJ* 2018;**360**: k764.

7. Afshar N, English DR, Blakely T, Thursfield V, Farrugia H, Giles GG, Milne RL. Differences in cancer survival by area-level socio-economic disadvantage: a population-based study using cancer registry data. *PLOS One* 2020;**15**: e0228551.

8. Antunes L, Mendonça D, Bento MJ, Rachet B. No inequalities in survival from colorectal cancer by education and socioeconomic deprivation - a population-based study in the North Region of Portugal, 2000-2002. *BMC Cancer* 2016;**16**: 608.

9. Woods LM, Rachet B, O'Connell D, Lawrence G, Coleman MP. Impact of deprivation on breast cancer survival among women eligible for mammographic screening in the West Midlands (UK) and New South Wales (Australia): Women diagnosed 1997-2006. *Int J Cancer* 2016;**138**: 2396-403.

10. Nuñez O, Rodríguez Barranco M, Fernández-Navarro P, Redondo Sanchez D, Luque Fernández M, Pollán Santamaría M, Sánchez MJ. Deprivation gap in colorectal cancer survival attributable to stage at diagnosis: a population-based study in Spain. *Cancer Epidemiol* 2020;**68**: 101794.

11. Ellis L, Coleman MP, Rachet B. How many deaths would be avoidable if socioeconomic inequalities in cancer survival in England were eliminated? A national population-based study, 1996-2006. *Eur J Cancer* 2012;**48**: 270-8.

12. NHS England. *NHS Long Term Plan.* (2020). https://www.longtermplan.nhs.uk/

13. Department of Health. *The NHS Plan: a plan for investment, a plan for reform.* (2000). https://webarchive.nationalarchives.gov.uk/20130124064356/http://www.dh.gov.uk/prod_consum_dh/groups/dh_digitalassets/@dh/@en/@ps/documents/digitalasset/dh_118522.pdf

14. Department of Health. *Cancer Reform Strategy.* (2007). https://www.nhs.uk/NHSEngland/NSF/Documents/Cancer%20Reform%20Strategy.pdf

15. Department of Health. *Improving Outcomes: a strategy for cancer.* (2010). https://assets.publishing.service.gov.uk/government/uploads/system/uploads/attachment_data/file/213785/dh_123394.pdf

16. McCutchan GM, Wood F, Edwards A, Richards R, Brain KE. Influences of cancer symptom knowledge, beliefs and barriers on cancer symptom presentation in relation to socioeconomic deprivation: a systematic review. *BMC Cancer* 2015;**15**: 1000.

17. Woods LM, Rachet B, Coleman MP. Choice of geographic unit influences socioeconomic inequalities in breast cancer survival. *Br J Cancer* 2005;**92**: 1279-82.

18. Quaglia A, Lillini R, Mamo C, Ivaldi E, Vercelli M, Group SW. Socio-economic inequalities: a review of methodological issues and the relationships with cancer survival. *Crit Rev Oncol Hematol* 2013;**85**: 266-77.

19. Niksic M, Rachet B, Warburton FG, Wardle J, Ramirez AJ, Forbes LJ. Cancer symptom awareness and barriers to symptomatic presentation in England - are we clear on cancer? *Br J Cancer* 2015;**113**: 533-42.

20. Jansen L, Behrens G, Finke I, Maier W, Gerken M, Pritzkuleit R, Holleczek B, Brenner H. Area-based socioeconomic inequalities in colorectal cancer survival in Germany: investigation based on population-based clinical cancer registration. *Front Oncol* 2020;**10**: 857.

21. Rosskamp M, Verbeeck J, Sass V, Gadeyne S, Verdoodt F, De Schutter H. Social inequalities in cancer survival in Belgium: a population-based cohort study. *Cancer Epidemiol Biomarkers Prev* 2021;**30**: 45-52.

22. Woods LM, Rachet B, Coleman MP. Origins of socio-economic inequalities in cancer survival: a review. *Ann Oncol* 2006;**17**: 5-19.

23. Bergin RJ, Emery J, Bollard RC, Falborg AZ, Jensen H, Weller D, Menon U, Vedsted P, Thomas RJ, Whitfield K, White V. Rural-urban dsparities in time to diagnosis and treatment for colorectal and breast cancer. *Cancer Epidemiol Biomarkers Prev* 2018;**27**: 1036-46.

24. Weber M. Class status and power. In: Gerth H, Mills CW. *From Max Weber: essays in sociology*ed. New York: Oxford University Press, 1946.

25. Ingleby FC, Belot A, Atherton I, Baker M, Elliss-Brookes L, Woods LM. Assessment of the concordance between individual-level and area-level measures of socio-economic deprivation in a cancer patient cohort in England and Wales. *BMJ Open* 2020;**10**: e041714.

26. Henson KE, Elliss-Brookes L, Coupland VH, Payne E, Vernon S, Rous B, Rashbass J. Data Resource Profile: National Cancer Registration Dataset in England. *International Journal of Epidemiology* 2019;**49**: 16-h.

27. Hattersley L, Creeser R, Longitudinal Study 1971-1991: History, organisation and quality of data. Office of Population Censuses and Surveys, 1995.

28. Shelton N, Marshall CE, Stuchbury R, Grundy E, Dennett A, Tomlinson J, Duke-Williams O, Xun W. Cohort Profile: the Office for National Statistics Longitudinal Study (The LS). *International Journal of Epidemiology* 2018;**48**: 383-4g.

29. Ministry of Housing, Communities & Local Government. *English indices of deprivation.* (2020). https://www.gov.uk/government/collections/english-indices-of-deprivation

For peer review only

30. Welsh Government. *Welsh index of multiple deprivation.* (2020). https://gov.wales/welsh-index-multiple-deprivation

31. Clemens T, Dibben C. A method for estimating wage, using standardised occupational classifications, for use in medical research in the place of self-reported income. *BMC Medical Research Methodology* 2014;**14**: 59.

32. Office for National Statistics. *The national statistics socio-economic classification (NS-SEC).* (2020). https://www.ons.gov.uk/methodology/classificationsandstandards/otherclassifications/thenationalstatisticssocioeconomicclassificationnssecrebasedonsoc2010

33. Perme MP, Stare J, Esteve J. On estimation in relative survival. *Biometrics* 2012;**68**: 113-20.

34. Perme MP, Pavlic K. Nonparametric relative survival analysis with the R package relsurv. *2018* 2018;**87**: 27.

35. R, ed. 3.6.3.

36. Ingleby FC, Woods LM, Atherton IM, Baker M, Elliss-Brookes L, Belot A. Describing socio-economic variation in life expectancy according to an individual's education, occupation and wage in England and Wales: an analysis of the ONS Longitudinal Study. *SSM Popul Health* 2021;**14**: 100815.

37. Inequalites in Cancer Outcomes Network. *Life tables for cancer survival analyses.* https://icon.lshtm.ac.uk/life-tables/

For peer review only

Table 1. Distribution of cancer patients in analysis cohort (N and %) compared to distribution (%) in England & Wales, patients diagnosed 1 January 2008 and 30 April 2016, by age group, cancer site, and sex.

Cancer	Men			Women		
	ONS-LS N	ONS-LS %	E+W* %	ONS-LS N	ONS-LS %	E+W* %
Breast						
20-54				1050	30.3	32.0
55-64				812	23.3	23.1
65-74				834	24.0	21.2
75+				777	22.4	23.7
Total				*3473*	*100.0*	*100.0*
Prostate						
20-54	109	3.6	3.9			
55-64	624	20.5	21.0			
65-74	1240	40.6	39.0			
75+	1071	35.2	36.1			
Total	*3044*	*100.0*	*100.0*			
Colorectal						
20-54	142	9.3	9.4	130	10.5	11.0
55-64	325	21.4	20.1	233	18.8	17.0
65-74	486	31.9	31.8	343	27.7	25.9
75+	569	37.4	38.7	531	43.0	46.1
Total	*1522*	*100.0*	*100.0*	*1237*	*100.0*	*100.0*
NHL						
20-54	90	18.6	19.7	71	19.0	16.7
55-64	108	22.4	19.8	62	16.7	18.7
65-74	141	29.2	27.9	110	29.6	27.3
75+	144	29.8	32.6	129	34.7	37.3
Total	*483*	*100.0*	*100.0*	*372*	*100.0*	*100.0*
Bladder						
20-54	32	6.4	4.9	15	7.4	5.6
55-64	80	15.9	13.6	19	9.4	11.4
65-74	160	31.9	30.1	61	30.2	24.1
75+	230	45.8	51.4	107	53.0	58.9
Total	*502*	*100.0*	*100.0*	*202*	*100.0*	*100.0*

*Data Sources: National Cancer Registry Data, ONS LS

Table 2. Number and percentage of men and women with each cancer type who died within 1 and 5 years of their diagnosis compared to England & Wales, patients diagnosed 1 January 2008 and 30 April 2016.

Cancer	Men					Women				
	ONS-LS N	ONS-LS % 1y	E+W* % 1y	ONS-LS % 5y	E+W* % 5y	ONS-LS N	ONS-LS % 1y	E+W* % 1y	ONS-LS % 5y	E+W* % 5y
Breast	-	-	-	-	-	3473	5%	6%	18%	21%
Prostate	3044	7%	8%	23%	27%	-	-	-	-	-
Colorectal	1522	23%	25%	47%	51%	1237	24%	28%	46%	45%
NHL	483	23%	25%	39%	43%	372	20%	22%	36%	40%
Bladder	502	25%	28%	51%	55%	202	39%	41%	56%	59%

*Data Sources: National Cancer Registry Data, ONS-LS

For peer review only - http://bmjopen.bmj.com/site/about/guidelines.xhtml

Figure legends

Figure 1. Age-standardised net survival (ASNS) estimates (95% CI) for individual- compared to area-level measures of **education**: patients diagnosed 2008-2016

a) Men
b) Women

Figure 2. Age-standardised net survival (ASNS) estimates (95% CI) for individual- compared to area-level measures of **income**: patients diagnosed 2008-2016

a) Men
b) Women

Figure 3. Age-standardised net survival (ASNS) estimates (95% CI) for individual- compared to area-level measures of **occupation**: patients diagnosed 2008-2016

a) Men
b) Women

Figure 4. Comparison of individual vs. area-level deprivation gaps[a]

Footnote to Figure 4:

[a]Deprivation gaps are negative where survival is lower in the more deprived groups. The dashed line indicates where the gap according to individual socio-economic group and the area-level index is equal (i.e. y=x).

Footnote required for all Figures 1-4

Data source: ONS LS

For peer review only

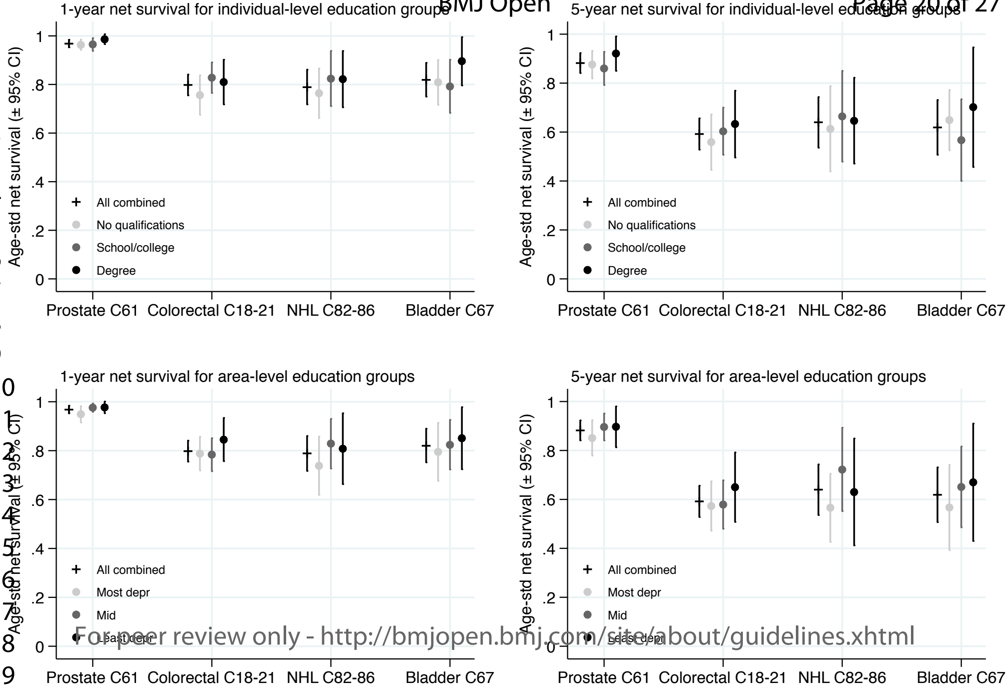

For peer review only - http://bmjopen.bmj.com/site/about/guidelines.xhtml

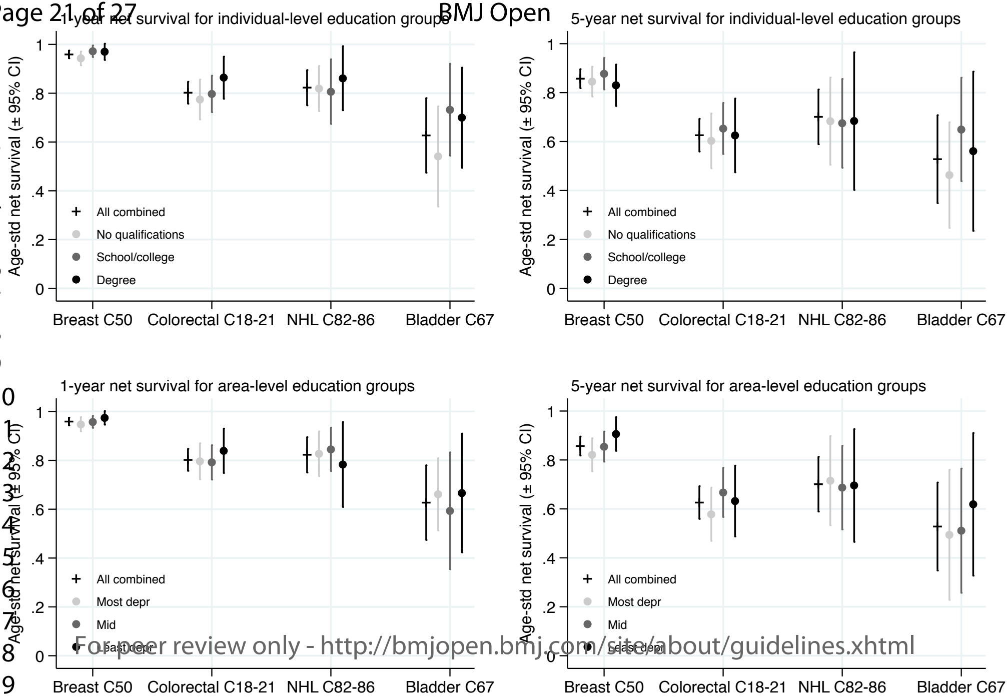

For peer review only - http://bmjopen.bmj.com/site/about/guidelines.xhtml

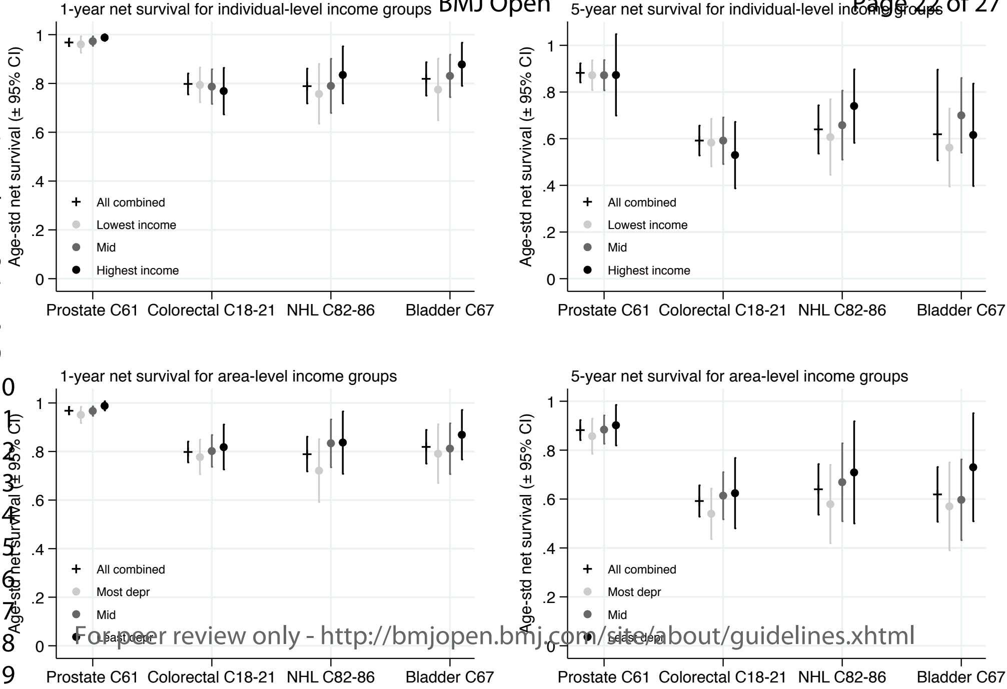
1-year net survival for individual-level income groups
5-year net survival for individual-level income groups
1-year net survival for area-level income groups
5-year net survival for area-level income groups
Age-std net survival (± 95% CI)
1
.8
.6
.4
.2
0
All combined
Lowest income
Mid
Highest income
Most depr
Least depr
Prostate C61
Colorectal C18-21
NHL C82-86
Bladder C67

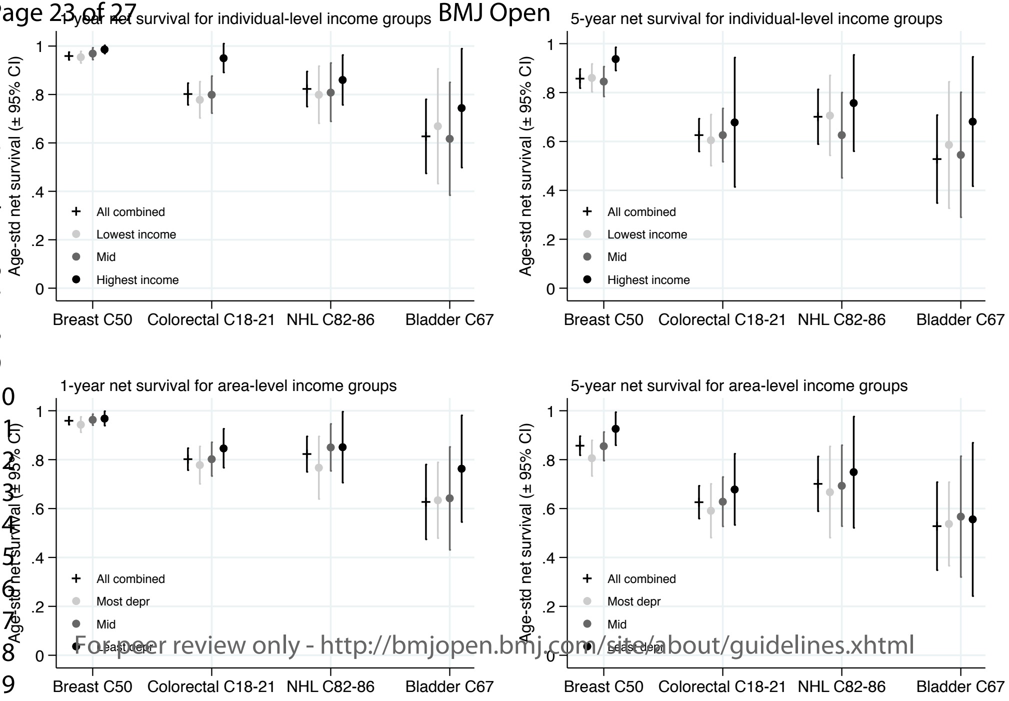

For peer review only - http://bmjopen.bmj.com/site/about/guidelines.xhtml

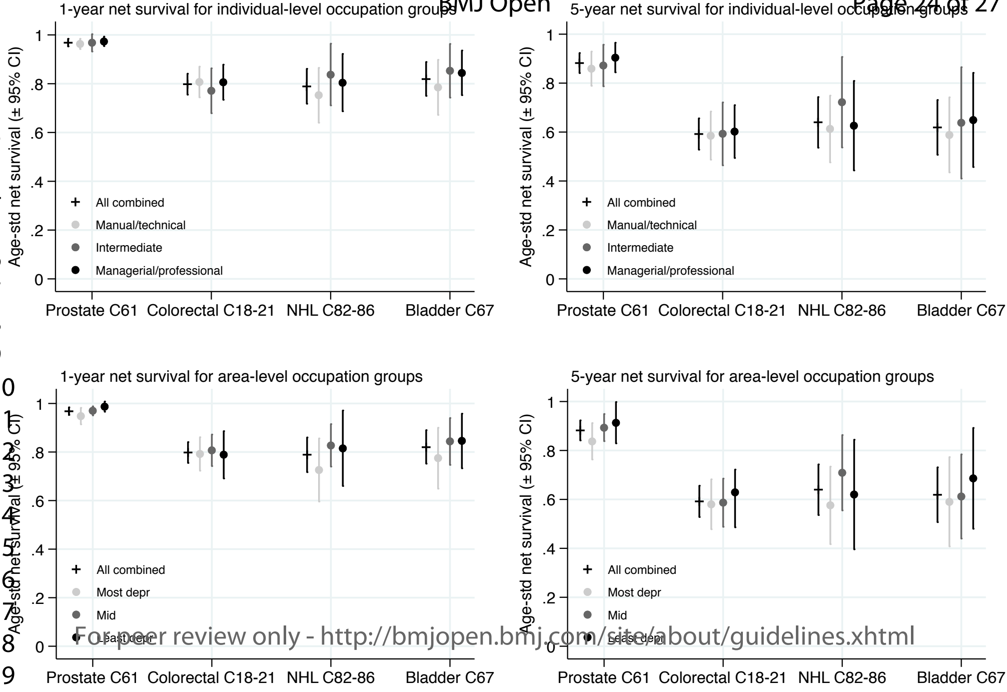

For peer review only - http://bmjopen.bmj.com/site/about/guidelines.xhtml

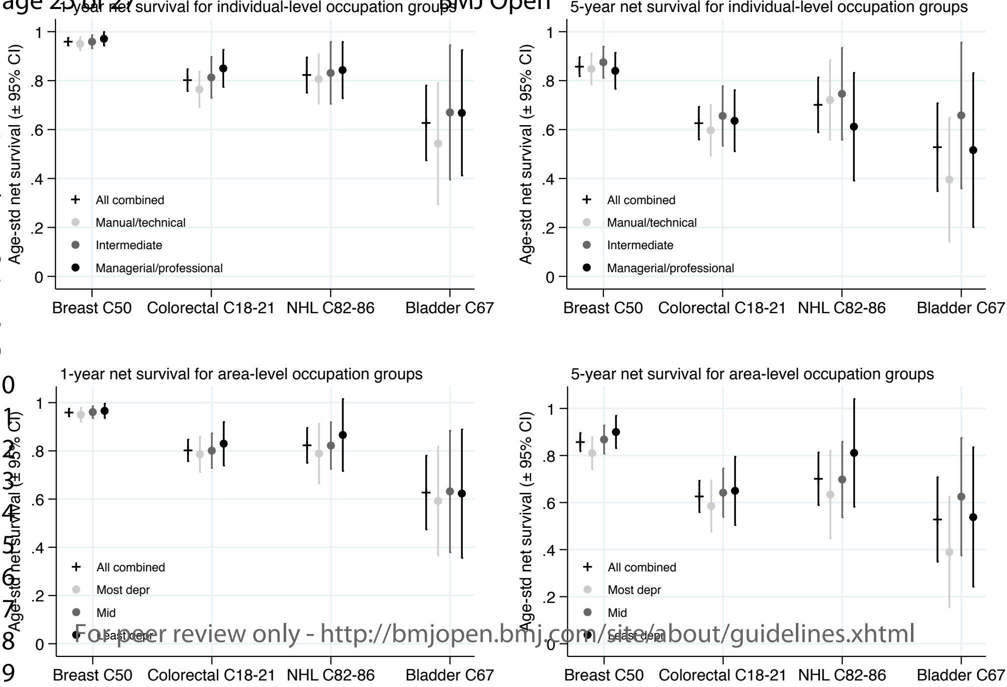

For peer review only - http://bmjopen.bmj.com/site/about/guidelines.xhtml

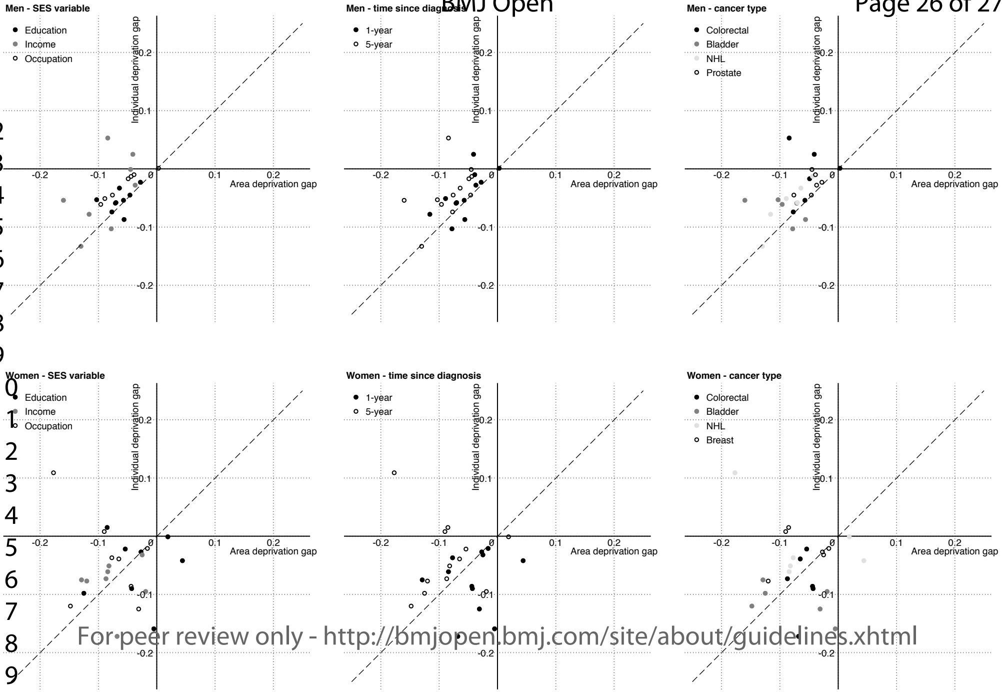
Men - SES variable
Education
Income
Occupation
Men - time since diagnosis
1-year
5-year
Men - cancer type
Colorectal
Bladder
NHL
Prostate
Women - SES variable
Education
Income
Occupation
Women - time since diagnosis
1-year
5-year
Women - cancer type
Colorectal
Bladder
NHL
Breast
Individual deprivation gap
Area deprivation gap
-0.2
-0.1
0
0.1
0.2

STROBE Statement—Checklist of items that should be included in reports of ***cohort studies***

	Item No	Recommendation	Page No
**Title and abstract**	1	(*a*) Indicate the study's design with a commonly used term in the title or the abstract	2
		(*b*) Provide in the abstract an informative and balanced summary of what was done and what was found	3
**Introduction**			
Background/rationale	2	Explain the scientific background and rationale for the investigation being reported	5
Objectives	3	State specific objectives, including any prespecified hypotheses	6
**Methods**			
Study design	4	Present key elements of study design early in the paper	6-7
Setting	5	Describe the setting, locations, and relevant dates, including periods of recruitment, exposure, follow-up, and data collection	6
Participants	6	(*a*) Give the eligibility criteria, and the sources and methods of selection of participants. Describe methods of follow-up	6, 7
		(*b*) For matched studies, give matching criteria and number of exposed and unexposed	N/A
Variables	7	Clearly define all outcomes, exposures, predictors, potential confounders, and effect modifiers. Give diagnostic criteria, if applicable	6, 7
Data sources/ measurement	8*	For each variable of interest, give sources of data and details of methods of assessment (measurement). Describe comparability of assessment methods if there is more than one group	6, 7
Bias	9	Describe any efforts to address potential sources of bias	
Study size	10	Explain how the study size was arrived at	6
Quantitative variables	11	Explain how quantitative variables were handled in the analyses. If applicable, describe which groupings were chosen and why	6, 7, 8
Statistical methods	12	(*a*) Describe all statistical methods, including those used to control for confounding	7, 8
		(*b*) Describe any methods used to examine subgroups and interactions	
		(*c*) Explain how missing data were addressed	
		(*d*) If applicable, explain how loss to follow-up was addressed	
		(*e*) Describe any sensitivity analyses	
**Results**			
Participants	13*	(a) Report numbers of individuals at each stage of study—eg numbers potentially eligible, examined for eligibility, confirmed eligible, included in the study, completing follow-up, and analysed	T1 T2
		(b) Give reasons for non-participation at each stage	
		(c) Consider use of a flow diagram	
Descriptive data	14*	(a) Give characteristics of study participants (eg demographic, clinical, social) and information on exposures and potential confounders	8, T1, T2
		(b) Indicate number of participants with missing data for each variable of interest	T1
		(c) Summarise follow-up time (eg, average and total amount)	T1
Outcome data	15*	Report numbers of outcome events or summary measures over time	T1, 8, 9

For peer review only - http://bmjopen.bmj.com/site/about/guidelines.xhtml

Main results	16	(*a*) Give unadjusted estimates and, if applicable, confounder-adjusted estimates and their precision (eg, 95% confidence interval). Make clear which confounders were adjusted for and why they were included<br>(*b*) Report category boundaries when continuous variables were categorized<br>(*c*) If relevant, consider translating estimates of relative risk into absolute risk for a meaningful time period	8, 9
Other analyses	17	Report other analyses done—eg analyses of subgroups and interactions, and sensitivity analyses	
**Discussion**			
Key results	18	Summarise key results with reference to study objectives	9, 10
Limitations	19	Discuss limitations of the study, taking into account sources of potential bias or imprecision. Discuss both direction and magnitude of any potential bias	11
Interpretation	20	Give a cautious overall interpretation of results considering objectives, limitations, multiplicity of analyses, results from similar studies, and other relevant evidence	10,11
Generalisability	21	Discuss the generalisability (external validity) of the study results	11, 12
**Other information**			
Funding	22	Give the source of funding and the role of the funders for the present study and, if applicable, for the original study on which the present article is based	14

*Give information separately for exposed and unexposed groups.

Note: An Explanation and Elaboration article discusses each checklist item and gives methodological background and published examples of transparent reporting. The STROBE checklist is best used in conjunction with this article (freely available on the Web sites of PLoS Medicine at http://www.plosmedicine.org/, Annals of Internal Medicine at http://www.annals.org/, and Epidemiology at http://www.epidem.com/). Information on the STROBE Initiative is available at http://www.strobe-statement.org.

For peer review only

For peer review only - http://bmjopen.bmj.com/site/about/guidelines.xhtml

BMJ Open

Are deprivation-specific cancer survival patterns similar according to individual- and area-based measures? A cohort study of patients diagnosed with five malignancies in England & Wales, 2008-2016

Journal:	*BMJ Open*
Manuscript ID	bmjopen-2021-058411.R1
Article Type:	Original research
Date Submitted by the Author:	06-Apr-2022
Complete List of Authors:	Woods, Laura; London School of Hygiene & Tropical Medicine, Department of Non-Communicable Disease Epidemiology<br>Belot, Aurelien; London School of Hygiene & Tropical Medicine<br>Atherton, Iain; Napier University SAFSL<br>Ellis-Brookes, Lucy; Public Health England<br>Baker, Matthew ; National Cancer Research Institute, Consumer Forum<br>Ingleby, Fiona; London School of Hygiene & Tropical Medicine, Department for Non-Communicable Disease Epidemiology
**Primary Subject Heading**:	Epidemiology
Secondary Subject Heading:	Oncology
Keywords:	Epidemiology < ONCOLOGY, Adult oncology < ONCOLOGY, PUBLIC HEALTH, QUALITATIVE RESEARCH

SCHOLARONE™
Manuscripts

I, the Submitting Author has the right to grant and does grant on behalf of all authors of the Work (as defined in the below author licence), an exclusive licence and/or a non-exclusive licence for contributions from authors who are: i) UK Crown employees; ii) where BMJ has agreed a CC-BY licence shall apply, and/or iii) in accordance with the terms applicable for US Federal Government officers or employees acting as part of their official duties; on a worldwide, perpetual, irrevocable, royalty-free basis to BMJ Publishing Group Ltd ("BMJ") its licensees and where the relevant Journal is co-owned by BMJ to the co-owners of the Journal, to publish the Work in this journal and any other BMJ products and to exploit all rights, as set out in our licence.

The Submitting Author accepts and understands that any supply made under these terms is made by BMJ to the Submitting Author unless you are acting as an employee on behalf of your employer or a postgraduate student of an affiliated institution which is paying any applicable article publishing charge ("APC") for Open Access articles. Where the Submitting Author wishes to make the Work available on an Open Access basis (and intends to pay the relevant APC), the terms of reuse of such Open Access shall be governed by a Creative Commons licence – details of these licences and which Creative Commons licence will apply to this Work are set out in our licence referred to above.

Other than as permitted in any relevant BMJ Author's Self Archiving Policies, I confirm this Work has not been accepted for publication elsewhere, is not being considered for publication elsewhere and does not duplicate material already published. I confirm all authors consent to publication of this Work and authorise the granting of this licence.

For peer review only

Research Paper

Are deprivation-specific cancer survival patterns similar according to individual- and area-based measures? A cohort study of patients diagnosed with five malignancies in England & Wales, 2008-2016

Laura M Woods*[1], Aurélien Belot[1], Iain Atherton[2], Lucy Ellis-Brookes[3], Mat Baker[4], Fiona C Ingleby[1]

1. Inequalities in Cancer Outcomes Network, Department of Non-Communicable Disease Epidemiology, Faculty of Epidemiology and Population Health, London School of Hygiene and Tropical Medicine, London, UK

2. School of Health & Social Care, Edinburgh Napier University, Edinburgh, UK

3. National Cancer Registration and Analysis Service, Public Health England, London, UK

4. National Cancer Research Institute Consumer Forum, London, UK

* Corresponding author:

laura.woods@lshtm.ac.uk

ORCID https://orcid.org/0000-0002-2178-1577

Twitter handle: @icon_lshtm

For peer review only

Abstract

Objective: To investigate if measured inequalities in cancer survival differ when using individual- (‘*person*’) compared to area- (‘*place*’) based measures of deprivation for three socio-economic dimensions: income, deprivation and occupation

Design: Cohort study

Setting: Data from the Office for National Statistics (ONS) Longitudinal Study of England and Wales, UK, linked to the National Cancer Registration Database

Participants: Patients diagnosed with cancers of the colorectum, breast, prostate, bladder or with Non-Hodgkin Lymphoma (NHL) during the period 2008-2016

Primary and secondary outcome measures: Differentials in net survival between groups defined by individual wage, occupation and education compared to those obtained from corresponding area-level metrics using the English and Welsh Indices of Multiple Deprivation (IMD).

Results: Survival was negatively associated with area-based deprivation irrespective of the type analysed, although a trend from least to most deprived was not always observed. Socio-economic differences were present according to individually-measured socio-economic groups although there was an absence of a consistent ‘gradient’ in survival. The magnitude of differentials was similar for area-based and individually-derived measures of deprivation, which was unexpected.

Conclusion: These unique data suggest that the socio-economic influence of ‘*person’* is different to that of ‘*place’* with respect to cancer outcomes. This has implications for health policy aimed at reducing inequalities. Further research could further consider the separate and additional influence of area-based deprivation over individual-level characteristics (contextual effects) as well as investigate the geographic, socio-economic and healthcare related characteristics of areas with poor outcomes in order to inform policy intervention.

For peer review only

Strengths and limitations of this study

- We analysed a unique, representative, cohort of England and Wales within which it was possible to classify individuals by both their area-based deprivation score and individual socioeconomic group.
- We used newly derived life tables for individual-level socioeconomic analyses to estimate underlying mortality for each individual socioeconomic group. We used a generic life table for area-based deprivation analyses because education- and occupation-specific life tables were unavailable, but also to prioritise the use of mortality rates derived from the ONS-LS itself.
- We estimated individual wage on the basis of recorded occupation due to the absence of directly measured data on earnings.
- Our study design enabled us to assess the relative impact of *'person'* vs. *'place'* upon socio-economic differentials in cancer outcomes.

For peer review only

Introduction

It has been widely documented that there are long-standing, persistent inequalities in cancer outcomes between individuals living in more deprived areas and those living in less deprived areas in higher income countries.[1-7] These inequalities may partly explain why cancer survival in the UK is lower than other similar settings, where socio-economic differentials tend to either be smaller or explicable by factors such as stage of disease at presentation.[8-10] The public health impact of these disparities is considerable as shown by the large number of associated avoidable deaths[11] and the influence this body of work has had on UK health policy over a period of more than twenty years.[12-15]

Despite their widespread use, the exact meaning of differentials observed between geographic areas with contrasting levels of deprivation have been variably interpreted. Most often, poorer outcomes amongst *'persons living in deprived areas'* has been treated as a proxy for poorer outcomes amongst *'deprived persons'*[16] without consideration that area-level deprivation could have a separate and independent influence over and above an individual's own personal characteristics ("contextual effect"). This has led in turn to an implicit assumption that the inequalities observed between affluent and deprived areas are most likely diluted versions of the 'real' (unknown) differences between individuals of different socio-economic groups, perhaps driven by the fact that, and consistent with the ecological fallacy, larger differentials are observed when the size of the geographical unit of analysis is smaller.[17,18]

Observed trends at the area-level combined with an inherent assumption of a dilution effect has thus tended to steer policy-related research into the individual domain, for example raising individual symptom awareness in these populations,[19] increasing the probability of early stage diagnosis through screening, and ensuring appropriate and effective treatment is given to patients living in more deprived localities.[19,20] Studies consistently documenting poorer outcomes in more deprived areas has also fuelled change in the way funding is allocated, causing them to be tied to specific assessments of unmet need, along with measurable, mostly individually-orientated, goals and mechanisms by which health inequalities might be narrowed.[12]

Only a relatively small body of research has examined cancer outcomes using individual, personal, measures of socio-economic status,[21] principally because data sources suitable for such an analysis are fewer and, or, more difficult to access.[22] Socio-economic differentials have relatively infrequently been considered as geographical phenomenon driven by locality-based factors such as travel time to hospital, access to GP services, except in especially rural settings outside the UK.[23] Similarly, the influence of community characteristics including social capital or social cohesion has not been widely considered. These social environmental influences on health outcomes, if important, are likely to be driven by a separate set of factors to those acting purely at an individual level.

For peer review only

Added to this, throughout the literature on inequalities there has tended to be a singular focus on a single dimension of deprivation, normally income, or a single composite score.[22] Relatively few studies have considered whether the different dimensions of deprivation have a similar or different effect, implicitly assuming that a single measure is sufficient to examine the underlying phenomenon of interest. A broader consideration of the relative contributions of wealth, status and power[24] upon cancer outcomes could help to clarify the mechanisms by which inequalities arise and are perpetuated.

Using the Office for National Statistics Longitudinal Study (ONS-LS) we have recently demonstrated that the concordance between individual socio-economic group and the deprivation present in the small area of residence is relatively low amongst cancer patients for three separate domains, but most especially for income.[25] These previous analyses suggest that interpreting area-based analyses derived from a single measure as broadly representative of individual inequalities therefore risks overlooking some important sub-groups of individuals. The objective of this follow-up study was to quantify and compare inequalities according to individual and area-based measures, contrasting the impact of income, occupation and education upon survival.

Methods

Cancer patient cohort

We analysed records from the national cancer registry[26] individually linked to the Office for National Statistics Longitudinal Study (LS).[27] The LS sample is a random sample clustered by date of birth.[25,28] Census data for cohort members are available from the 1971 census through to the 2011 census. The ONS LS also links life events data, including cancer registrations and deaths of members. The analysis cohort for this study included LS members present at either or both 2001 and 2011 census, and diagnosed with a first primary malignant cancer diagnosis between 1 January 2008 and 30 April 2016 at ages 20-100 years old. We examined five common cancer types: breast (ICD-10 code C50), prostate (C61), colorectal (C18-21), Non-Hodgkin Lymphoma (C82-86), and bladder (C67). These specific malignancies were selected as it has been demonstrated that they exhibit significant area-based socio-economic differentials for both sexes.[5] A small number (<20) of sex-site errors, and also a small number (<30) of men with breast cancer were excluded.

Area-level deprivation

The Indices of Multiple Deprivation for England[29] and Wales[30] were used to measure area-based deprivation. We used the income, employment (i.e., occupation) and education domains for the Lower Super Output Area of residence, using the temporally closest score to each census. For the 2001 census, this was the English IMD2004 and the Welsh metrics reported in 2005. For the 2011 census, this was the English IMD2015 and the Welsh metrics reported in 2014. Each index was linked to the data as quintiles of the national distribution

For peer review only

of areas, and three deprivation groups were created for the purposes of this analysis: least deprived (quintiles 1 & 2), mid (quintile 3) and most deprived (quintiles 4 & 5).

Individual-level socio-economic variables

Individual data on age, sex, qualifications and occupation were extracted directly from census data for each patient. Occupation type was derived using the 3-group version of the National Statistics Socio-Economic Classification (NS-SEC) to ensure sufficient numbers to enable statistical analysis. These are “technical, routine and manual occupations”; “intermediate occupations”; or “higher managerial, administrative and professional occupations”.[29]

Education level was categorised as one of three groups based on standard levels of English and Welsh qualifications used in the census: “no qualifications”; “school or college qualifications (GCSEs, A-levels, apprenticeships, vocational qualifications or equivalent)”; or “degree qualifications (degree-level education or higher)”.

Individual weekly income (GBP) was estimated indirectly from census data on an individual’s age, sex, and Standard Occupational Classification (SOC) code using an externally-validated linear model prediction method described by Clemens and Dibben.[31] We took a data-driven approach to adjust income for those aged over 60, who were most likely to be retired. We adjusted these income estimates using the observed annualised percentage decreases in income for those aged over 60 reported by the English Longitudinal Study of Ageing.[32] After applying this correction, income estimates were grouped into quintiles separately for each sex. LS members were then categorised into three groups by estimated income: lowest income (quintiles 1 & 2), middle income (quintile 3) and highest income (quintiles 4 & 5). Quintiles were calculated based on all available LS members (i.e., not just cancer patients), separately for each sex. Income estimates were therefore linked to occupation, however, the use of SOC codes rather than NS-SEC (as for the occupation variable above) means that these variables are independent of one another, since SOC codes are linked to specific jobs, as opposed to the broad NS-SEC categories for types of occupation.

Data were not available from the 2011 census for a small proportion of individuals; mostly accounted for by those who were diagnosed with cancer between 2008-2011 and died prior to the 2011 census.[25] Where possible, data from the 2001 census were used for these individuals. Missing data on qualifications or occupation (which includes long-term unemployed and students for the 3-group version of the NS-SEC as recommended by the NS-SEC guidelines[29]), were completed where possible by proxy, using another adult resident in the household (usually household head). Following this procedure, 6%, <1%, and 5% of records were missing individual deprivation data for occupation, education, and income respectively. These individuals were excluded.

For peer review only

Survival analysis

Analyses were carried out separately for men and women. We analysed survival time (days between date of diagnosis and date of death or censoring) as a function of patient age and either socio-economic group or area-based deprivation group, adjusted for the 'expected' mortality. Data were censored on the 31-Dec-2017, the date of the most recent linkage of the ONS-LS to mortality records.

We report net survival and 95% confidence intervals, calculated using the non-parametric Pohar-Perme estimator[33] with the 'relsurv'[34] package in R v.3.6.3.[35] This is the most widely used, consistent estimator of net survival. Net survival is the survival probability patients would experience if their only possible cause of death were cancer. Net survival estimates are independent of underlying other-cause mortality and thus reflect cancer-specific prognosis. We account for underlying deaths from other causes using 'expected' mortality estimates for each individual socio-economic group, which we extracted from life tables that we derived from this same ONS-LS cohort.[36] Expected mortality for the area-level deprivation analyses used life tables based on the overall ONS-LS cohort.

Net survival is reported as age-standardised estimates (Age-Standardised Net Survival, ASNS), derived using ICSS weights[37] for age groups, with the youngest two groups merged together (i.e. 15-54, 55-64, 65-74, 75+) to allow for the lower numbers in the youngest age groups in this population sample. For each deprivation or socio-economic status measure and each cancer type, we calculated the arithmetic difference in survival between the most affluent and most deprived groups as the 'survival gap' (irrespective of which group displayed the highest or lowest survival).

Clustering of patients within geographical areas (LSOAs) was possible within the data, implying the need to take this into account in the analysis. However, an initial review of the data showed that a single-level analysis approach was sufficient here, as almost all individuals were unique to their LSOA within each cancer site. This applied to 96% of the analysis cohort; with the remaining 4% at a maximum of two individuals in the same geographic area. We therefore adopted a single-level approach.

Patient and Public Involvement

This study was first presented to patient representatives at the NCRI Consumer Forum 'Dragon's Den' in 2017, where Mr Matthew Baker, along with nine other members of the public, provided input and ideas for the approach and methodology. Mr Baker has acted as Non-Academic Co-Investigator, helped to develop the study protocol and implement the research plans. He has attended all project meetings to provide insights on decision making as the project progressed. Following the production of results the whole project team have worked on the dissemination and discussion of the results at both a further Dragon's Den meeting, and in online forums with policy makers. The research will also been presented online to members of the public who engaged with the topic via a specifically-targeted Facebook marketing campaign.

For peer review only

Results

Overall, 5,551 men and 5,284 women were included in the analyses. The cohort was broadly representative of the population from which it was drawn: the sex-specific age distribution of cases for each cancer site are similar to that of the overall population of England and Wales (Table 1). The data included a sufficiently large number of deaths by cancer and sex to enable net survival estimation (Table 2). Similar proportions of men and women died within 1 and 5 years of diagnosis in the ONS-LS and in England and Wales.

Socio-economic variations in net survival were observed at both 1 and 5 years after diagnosis for both sexes and for each cancer site. These results are displayed in Figures 1-3. Survival tended to be negatively associated with area-level deprivation irrespective of the type analysed, with estimates in the most deprived areas between 0.5% and 12.9% lower than in the least deprived areas at 1 year since diagnosis, and between 1.9% and 17.7% lower at 5 years. The only exceptions were for women with NHL, where area-based survival was not associated with increasing deprivation, and for men with colorectal cancer across occupation at 1-year. Differences across area-based income measures tended to show the most consistent and strongest negative associations. Patterns according to individual socio-economic group were more mixed. The association between survival and deprivation was generally weaker for occupation than for other types of socio-economic variables amongst men and, to a lesser extent, women. Percentage point differences in ASNS 1 year after diagnosis between individuals with degree-level qualifications and no qualifications ranged from 2.3% to 15.9%, amongst those with the highest and lowest incomes from -2.5% to 17.2%, and from -0.1% to 12.5% between those working in manual compared to professional occupations.

Differentials between individual-level socio-economic groups in comparison to area-based deprivation quintiles are plotted against one another as the 'survival gap' in Figure 4. The diagonal line indicates an equal extent of survival inequality measured in individual-level and area-level analysis. For men with colorectal and prostate cancer, the deprivation 'gap' was of a similar or slightly smaller magnitude between individual socio-economic groups compared to area-based quintiles, for both 1-and 5-year survival, for education, occupation and, to a lesser extent, income. Colorectal cancer differentials amongst women were greater using individual-based measures than area-based measures 1 year after diagnosis, but more similar 5 years after diagnosis, for all three types of deprivation. Breast cancer inequalities were of a similar magnitude 1 year after diagnosis for all types of deprivation, but larger for area-based measures after 5 years in comparison to those observed between individual socio-economic groups. The deprivation gap tended to be smallest overall for men with prostate cancer and, to a lesser extent, women with breast cancer.

Bladder and NHL are lower incidence malignancies so the number of cases and deaths we examined were much smaller (Tables 1 and 2). As such, the survival estimates for these cancers have wider confidence intervals and the interpretation of these data should be treated with caution. Amongst men patterns for NHL were similar to the more common cancer sites. Amongst women with NHL an unexpected reverse trend was

For peer review only

seen between area-based educational deprivation and survival, where more deprived women had better outcomes. More deprived bladder cancer patients displayed poorer outcomes amongst both men and women. There was a suggestion that area-based measures had a greater impact compared to individual socio-economic group for men with bladder cancer, but patterns for women were similar between area-based and individual measures.

Discussion

We have described the differences in non-parametric univariable net survival for five cancers previously shown to have substantial area-level deprivation gaps in survival,[5] comparing inequalities derived using area-based deprivation measures to those obtained using individual measures of socioeconomic status. Consistent with the literature, survival was most often lower amongst those from more deprived localities irrespective of the type of deprivation analysed.[6,17] By contrast, there was an unexpected lack of overall trend of lower survival across the spectrum of individual socio-economic groups as well as a notable lack of trend between individual income groups. Our results thus suggest that the role of individual characteristics ('*person*') versus area-based characteristics ('*place*') differs with respect to cancer outcomes and that the underlying reasons for this warrant further investigation.

Individual vs. area-based differentials

We calculated deprivation gaps in cancer survival in order to evaluate whether differentials between deprived and affluent individuals were larger, smaller, or similar to those between deprived and affluent populations. The similarity of the magnitude of the deprivation gaps across area- and individual-based measures suggests no evidence for a dilution effect, which was unexpected. Rather, these data are more supportive of the existence of two separate effects for cancer outcomes, one of '*person'* (individual effect), another of '*place'* (area-based effect). Our results are consistent with our previous findings which showed that deprived persons frequently resided in non-deprived areas,[25] and speaks against interpretations of area-based data where poorer health outcomes amongst deprived populations have been assumed to arise simply from poorer outcomes amongst deprived persons (dilution effect). The exception to this pattern is women's 1-year survival, where there is some suggestion of dilution for bladder and colorectal cancer, although these data points had wide confidence intervals. The smaller differentials we observed for prostate and breast cancers are likely in part to constitute a form of ceiling effect, since differentials tend to be smaller when survival is high, even if the excess hazard ratio is of a similar magnitude to other cancers with lower survival.

Domains of deprivation

For the most part the different measures of deprivation (income, education, occupation) exerted a broadly similar effect on cancer outcomes in area-based analyses. This has been previously observed[17] and is also consistent with sociological theory which states that socio-economic status arises from three inter-related domains: a person's social class (broadly reflected by attained educational level), their social status or marker

For peer review only

of prestige (broadly seen in the variety of occupations) and power (higher incomes affording a greater ability to spend and thus greater influence).[24] At the individual level, cancer outcomes were correlated with individual occupation and education for both men and women. Higher individual income amongst men was counter-intuitively associated with poorer outcomes in some analyses, and in others displayed no discernible trend. These results were somewhat unexpected, especially given the clear association observed between area-based income deprivation and cancer outcomes in these same patients. These observations could be explained in part by the imputation of individual income from occupational codes. Alternatively, it is possible that this is a threshold effect, where variations in income above a certain level are not strongly associated with cancer outcomes. This suggests that the existing literature on income deprivation patterns may be picking out differentials between populations with differing proportions of persons on very low incomes.

Strengths and limitations

Our study has a number of strengths. First, we used a unique, representative cohort of England and Wales within which we were able to classify individuals by both area-based deprivation score and individual socioeconomic group, so as to assess area- and individual-level patterns within the same cohort of patients. We used newly derived individual life tables[36] to estimate underlying mortality for each individual socioeconomic group, matched to the most up-to-date methodology for estimating non-parametric survival from cancer.[33] For prostate, breast and colorectal cancers we had data with sample sizes sufficiently large to generate relatively narrow confidence intervals around our point estimates and so reasonably compare outcomes and the 'survival gaps' between the two different approaches. Numbers for bladder cancer and non-Hodgkin lymphoma were smaller (smallest group 202 cases with approximately 80 deaths) and the confidence intervals much wider. These results should be interpreted with more caution: patterns for bladder cancer lent weight to our overall conclusions, whilst those for NHL were less consistent. Limitations to our approach include the use of a generic life table for the area-based analyses, as well as the need to estimate individual income on the basis of recorded occupation. Although life tables for England and Wales as a whole derived from quintiles of area-based income deprivation are available,[38] these have not been derived specifically for education and occupation sub-domains. Further, our methods prioritised using life tables derived from the same cohort, so as not to introduce a bias from the use of a national life table: whilst the LS is representative of the overall population it is still only a small sample of the whole of England and Wales combined, and so it was more appropriate to extract observed rates of death from the cohort itself. The income variable for individuals was necessarily an estimate, since this information is not directly collected in the UK Census. However, we used an externally-validated method,[31] which was based on a separate measure of occupation[29] to the employment domain, as well as age and sex, in order to generate the most accurate estimate as possible.

For peer review only

Comparisons with published literature

Although there are now a substantially greater number of analyses which examine the impact of individual measures of socio-economic status upon cancer survival,[39] particularly from the Nordic countries, only two recent studies have described the impact of individual vs. area-based measures on cancer survival as we do. In these studies, socio-economic differentials according to individual and neighbourhood measures amongst men diagnosed with prostate cancer[40] and women diagnosed with breast cancer,[41] in the USA are similar to each other, supporting our own conclusions for these patients diagnosed in the UK.

Policy implications & further research

Our results have significance for public health policy on inequalities, demonstrating that there is unlikely to be a simple correspondence between reducing differentials between more and less-deprived areas and improving outcomes for individually-deprived persons. Whilst our analyses of these same data demonstrate evidence for contextual effects in some groups,[42] further research is required to establish the mechanisms by which these patterns arise. In particular, whilst area-based measures are exactly that, based upon areas rather than individuals, they are derived from observed proportions of individual people experiencing or having specific personal characteristics of low socio-economic status within those areas (for example, the proportion of the population on income benefits, who are unemployed, or lack of formal qualifications). As such, they measure both the influence of individual characteristics and reflect something of those individual's context, whilst they do not include any environmental measures of deprivation such as access to services, travel time, travel costs, number of GPs or specialist oncologists per capita. Nor do they take into account the way that healthcare is organised and delivered in different settings which is likely to be influential.[43] A further consideration is the social or community setting: our results do not and cannot measure factors such as social capital or social cohesion which may also be influential on an individual's ability to access the healthcare available to them. More detailed analyses are therefore required to better understand area-based patterns: first to further understand the nature and importance of '*place'* in comparison to the influence of the socioeconomic status of the '*person*', specifically as determined by the social, geographical and healthcare characteristics of areas in contrast to the characteristics of the individuals residing in them, and second to investigate the particular intrinsic characteristics of areas associated with poorer outcomes.

Conclusion

We have conducted a unique analysis of cancer survival with respect to individual and area-based measures of deprivation. These data suggest that the influence of '*person'* and '*place'* upon cancer outcomes warrants further investigation as part of a public health strategy to reduce cancer, as well as wider health, inequalities. These data support our other analyses on the differential effect of area, over and above the individual, as well as the particular characteristics of areas with poor outcomes would enable the derivation of more

For peer review only

accurate hypotheses about the underlying causes of inequalities, and elucidate potential avenues for policy intervention.

For peer review only

Additional information

Acknowledgements

The permission of the Office for National Statistics to use the Longitudinal Study is gratefully acknowledged, as is the help provided by staff of the Centre for Longitudinal Study Information & User Support (CeLSIUS). CeLSIUS is supported by the ESRC Census of Population Programme under project ES/V003488/1. The authors alone are responsible for the interpretation of the data in this paper. This work uses data provided by patients and collected by the NHS as part of their care and support. Using patient data is vital to improve health and care for everyone. There is huge potential to make better use of information from people's patient records, to understand more about disease, develop new treatments, monitor safety, and plan NHS services. Patient data should be kept safe and secure, to protect everyone's privacy, and it's important that there are safeguards to make sure that it is stored and used responsibly. Everyone should be able to find out about how patient data is used.

Authors' contributions

LW, AB and IA conceived the study, developed the proposal, acquired the funding, ethical approval and data. They co-managed the conduct of the research and interpretation of the results. FI conducted all analyses, assisted with the interpretation of the data and drafted the initial manuscript. LW wrote the final version and led on the manuscript submission. LEB and MB assisted in the acquisition of funding, the interpretation of the data, and commented on drafts of the manuscript. All authors have approved the final version and agree to be accountable for all aspects of this work.

Ethics approval

Ethics approval for this study was obtained from the London School of Hygiene and Tropical Medicine Ethics Online Application 14600; approved 01/02/2018. Data presented for England and Wales and presented in Tables 1 and 2 were obtained following statutory approval from the Confidentiality Advisory Group (CAG) of the Health Research Authority (HRA): PIAG 1–05(c) 2007.

Data availability

Data are not publicly available but can be accessed via appropriate application to the ONS Longitudinal Study (https://www.ucl.ac.uk/epidemiology-health-care/research/epidemiology-and-public-health/research/health-and-social-surveys-research-group/studies-44). This work contains statistical data from ONS which is Crown Copyright. The use of the ONS statistical data in this work does not imply the endorsement of the ONS in relation to the interpretation or analysis of the statistical data. This work uses research datasets which may not exactly reproduce National Statistics aggregates. Data presented for England and Wales in Tables 1 and 2 are available via application to the Public Health England Office

For peer review only

for Data Release (https://www.gov.uk/government/publications/accessing-public-health-england-data/about-the-phe-odr-and-accessing-data).

Competing interests

The authors declare no competing interests.

Funding information

This work was supported by the Economic and Social Research Council (ES/S001808/1).

For peer review only

References

1. Coleman MP, Rachet B, Woods LM, Mitry E, Riga M, Cooper N, Quinn MJ, Brenner H, Esteve J. Trends and socioeconomic inequalities in cancer survival in England and Wales up to 2001. *Br J Cancer* 2004;**90**: 1367-73.

2. Finke I, Behrens G, Maier W, Schwettmann L, Pritzkuleit R, Holleczek B, Kajuter H, Gerken M, Mattutat J, Emrich K, Jansen L, Brenner H, German Cancer Survival Working G. Small-area analysis on socioeconomic inequalities in cancer survival for 25 cancer sites in Germany. *Int J Cancer* 2021.

3. Fowler H, Belot A, Njagi EN, Luque-Fernandez MA, Maringe C, Quaresma M, Kajiwara M, Rachet B. Persistent inequalities in 90-day colon cancer mortality: an English cohort study. *Br J Cancer* 2017;**117**: 1396-404.

4. Luque-Fernandez MA, Redondo-Sanchez D, Rodriguez-Barranco M, Chang-Chan YL, Salamanca-Fernandez E, Nunez O, Fernandez-Navarro P, Pollan M, Sanchez MJ. Socioeconomic inequalities in colorectal cancer survival in southern Spain: a multilevel population-based cohort study. *Clin Epidemiol* 2020;**12**: 797-806.

5. Rachet B, Ellis L, Maringe C, Chu T, Nur U, Quaresma M, Shah A, Walters S, Woods L, Forman D, Coleman MP. Socioeconomic inequalities in cancer survival in England after the NHS cancer plan. *Br J Cancer* 2010;**103**: 446-53.

6. Exarchakou A, Rachet B, Belot A, Maringe C, Coleman MP. Impact of national cancer policies on cancer survival trends and socioeconomic inequalities in England, 1996-2013: population based study. *BMJ* 2018;**360**: k764.

7. Afshar N, English DR, Blakely T, Thursfield V, Farrugia H, Giles GG, Milne RL. Differences in cancer survival by area-level socio-economic disadvantage: a population-based study using cancer registry data. *PLOS One* 2020;**15**: e0228551.

8. Antunes L, Mendonça D, Bento MJ, Rachet B. No inequalities in survival from colorectal cancer by education and socioeconomic deprivation - a population-based study in the North Region of Portugal, 2000-2002. *BMC Cancer* 2016;**16**: 608.

9. Woods LM, Rachet B, O'Connell D, Lawrence G, Coleman MP. Impact of deprivation on breast cancer survival among women eligible for mammographic screening in the West Midlands (UK) and New South Wales (Australia): Women diagnosed 1997-2006. *Int J Cancer* 2016;**138**: 2396-403.

10. Nuñez O, Rodríguez Barranco M, Fernández-Navarro P, Redondo Sanchez D, Luque Fernández M, Pollán Santamaría M, Sánchez MJ. Deprivation gap in colorectal cancer survival attributable to stage at diagnosis: a population-based study in Spain. *Cancer Epidemiol* 2020;**68**: 101794.

11. Ellis L, Coleman MP, Rachet B. How many deaths would be avoidable if socioeconomic inequalities in cancer survival in England were eliminated? A national population-based study, 1996-2006. *Eur J Cancer* 2012;**48**: 270-8.

12. NHS England. *NHS Long Term Plan.* (2020). https://www.longtermplan.nhs.uk/

13. Department of Health. *The NHS Plan: a plan for investment, a plan for reform.* (2000). https://webarchive.nationalarchives.gov.uk/20130124064356/http://www.dh.gov.uk/prod_consum_dh/groups/dh_digitalassets/@dh/@en/@ps/documents/digitalasset/dh_118522.pdf

14. Department of Health. *Cancer Reform Strategy.* (2007). https://www.nhs.uk/NHSEngland/NSF/Documents/Cancer%20Reform%20Strategy.pdf

15. Department of Health. *Improving Outcomes: a strategy for cancer.* (2010). https://assets.publishing.service.gov.uk/government/uploads/system/uploads/attachment_data/file/213785/dh_123394.pdf

16. McCutchan GM, Wood F, Edwards A, Richards R, Brain KE. Influences of cancer symptom knowledge, beliefs and barriers on cancer symptom presentation in relation to socioeconomic deprivation: a systematic review. *BMC Cancer* 2015;**15**: 1000.

17. Woods LM, Rachet B, Coleman MP. Choice of geographic unit influences socioeconomic inequalities in breast cancer survival. *Br J Cancer* 2005;**92**: 1279-82.

18. Quaglia A, Lillini R, Mamo C, Ivaldi E, Vercelli M, Group SW. Socio-economic inequalities: a review of methodological issues and the relationships with cancer survival. *Crit Rev Oncol Hematol* 2013;**85**: 266-77.

19. Niksic M, Rachet B, Warburton FG, Wardle J, Ramirez AJ, Forbes LJ. Cancer symptom awareness and barriers to symptomatic presentation in England - are we clear on cancer? *Br J Cancer* 2015;**113**: 533-42.

20. Jansen L, Behrens G, Finke I, Maier W, Gerken M, Pritzkuleit R, Holleczek B, Brenner H. Area-based socioeconomic inequalities in colorectal cancer survival in Germany: investigation based on population-based clinical cancer registration. *Front Oncol* 2020;**10**: 857.

21. Rosskamp M, Verbeeck J, Sass V, Gadeyne S, Verdoodt F, De Schutter H. Social inequalities in cancer survival in Belgium: a population-based cohort study. *Cancer Epidemiol Biomarkers Prev* 2021;**30**: 45-52.

22. Woods LM, Rachet B, Coleman MP. Origins of socio-economic inequalities in cancer survival: a review. *Ann Oncol* 2006;**17**: 5-19.

23. Bergin RJ, Emery J, Bollard RC, Falborg AZ, Jensen H, Weller D, Menon U, Vedsted P, Thomas RJ, Whitfield K, White V. Rural-urban dsparities in time to diagnosis and treatment for colorectal and breast cancer. *Cancer Epidemiol Biomarkers Prev* 2018;**27**: 1036-46.

24. Weber M. Class status and power. In: Gerth H, Mills CW. *From Max Weber: essays in sociology*ed. New York: Oxford University Press, 1946.

25. Ingleby FC, Belot A, Atherton I, Baker M, Elliss-Brookes L, Woods LM. Assessment of the concordance between individual-level and area-level measures of socio-economic deprivation in a cancer patient cohort in England and Wales. *BMJ Open* 2020;**10**: e041714.

26. Henson KE, Elliss-Brookes L, Coupland VH, Payne E, Vernon S, Rous B, Rashbass J. Data Resource Profile: National Cancer Registration Dataset in England. *International Journal of Epidemiology* 2019;**49**: 16-h.

27. Hattersley L, Creeser R, Longitudinal Study 1971-1991: History, organisation and quality of data. Office of Population Censuses and Surveys, 1995.

28. Shelton N, Marshall CE, Stuchbury R, Grundy E, Dennett A, Tomlinson J, Duke-Williams O, Xun W. Cohort Profile: the Office for National Statistics Longitudinal Study (The LS). *International Journal of Epidemiology* 2018;**48**: 383-4g.

29. Ministry of Housing, Communities & Local Government. *English indices of deprivation.* (2020). https://www.gov.uk/government/collections/english-indices-of-deprivation

For peer review only

30. Welsh Government. *Welsh index of multiple deprivation.* (2020). https://gov.wales/welsh-index-multiple-deprivation

31. Clemens T, Dibben C. A method for estimating wage, using standardised occupational classifications, for use in medical research in the place of self-reported income. *BMC Medical Research Methodology* 2014;**14**: 59.

32. Office for National Statistics. *The national statistics socio-economic classification (NS-SEC).* (2020). https://www.ons.gov.uk/methodology/classificationsandstandards/otherclassifications/thenationalstatisticssocioeconomicclassificationnssecrebasedonsoc2010

33. Perme MP, Stare J, Esteve J. On estimation in relative survival. *Biometrics* 2012;**68**: 113-20.

34. Perme MP, Pavlic K. Nonparametric relative survival analysis with the R package relsurv. *2018* 2018;**87**: 27.

35. R, ed. 3.6.3.

36. Ingleby FC, Woods LM, Atherton IM, Baker M, Elliss-Brookes L, Belot A. Describing socio-economic variation in life expectancy according to an individual's education, occupation and wage in England and Wales: an analysis of the ONS Longitudinal Study. *SSM Popul Health* 2021;**14**: 100815.

37. Corazziari I, Quinn M, Capocaccia R. Standard cancer patient population for age standardising survival ratios. *Eur J Cancer* 2004;**40**: 2307-16.

38. Inequalites in Cancer Outcomes Network. *Life tables for cancer survival analyses.* https://icon.lshtm.ac.uk/life-tables/

39. Afshar N, English DR, Milne RL. Factors Explaining Socio-Economic Inequalities in Cancer Survival: A Systematic Review. *Cancer Control* 2021;**28**: 10732748211011956.

40. DeRouen MC, Schupp CW, Koo J, Yang J, Hertz A, Shariff-Marco S, Cockburn M, Nelson DO, Ingles SA, John EM, Gomez SL. Impact of individual and neighborhood factors on disparities in prostate cancer survival. *Cancer Epidemiol* 2018;**53**: 1-11.

41. Sprague BL, Trentham-Dietz A, Gangnon RE, Ramchandani R, Hampton JM, Robert SA, Remington PL, Newcomb PA. Socioeconomic status and survival after an invasive breast cancer diagnosis. *Cancer* 2011;**117**: 1542-51.

42. Ingleby FC, Woods LM, Atherton IM, Baker M, Elliss-Brookes L, Belot A. An investigation of cancer survival inequalities associated with individual-level socio-economic status, area-level deprivation, and contextual effects, in a cancer patient cohort in England and Wales. *BMC Public Health* 2022;**22**: 90.

43. Burton C, O'Neill L, Oliver P, Murchie P. Contribution of primary care organisation and specialist care provider to variation in GP referrals for suspected cancer: ecological analysis of national data. *BMJ Qual Saf* 2020;**29**: 296-303.

For peer review only

Table 1. Distribution of cancer patients in analysis cohort (N and %) compared to distribution (%) in England & Wales, patients diagnosed 1 January 2008 and 30 April 2016, by age group, cancer site, and sex.

Cancer	Men			Women		
	ONS-LS N	ONS-LS %	E+W* %	ONS-LS N	ONS-LS %	E+W* %
Breast						
20-54				1050	30.3	32.0
55-64				812	23.3	23.1
65-74				834	24.0	21.2
75+				777	22.4	23.7
Total				*3473*	*100.0*	*100.0*
Prostate						
20-54	109	3.6	3.9			
55-64	624	20.5	21.0			
65-74	1240	40.6	39.0			
75+	1071	35.2	36.1			
Total	*3044*	*100.0*	*100.0*			
Colorectal						
20-54	142	9.3	9.4	130	10.5	11.0
55-64	325	21.4	20.1	233	18.8	17.0
65-74	486	31.9	31.8	343	27.7	25.9
75+	569	37.4	38.7	531	43.0	46.1
Total	*1522*	*100.0*	*100.0*	*1237*	*100.0*	*100.0*
NHL						
20-54	90	18.6	19.7	71	19.0	16.7
55-64	108	22.4	19.8	62	16.7	18.7
65-74	141	29.2	27.9	110	29.6	27.3
75+	144	29.8	32.6	129	34.7	37.3
Total	*483*	*100.0*	*100.0*	*372*	*100.0*	*100.0*
Bladder						
20-54	32	6.4	4.9	15	7.4	5.6
55-64	80	15.9	13.6	19	9.4	11.4
65-74	160	31.9	30.1	61	30.2	24.1
75+	230	45.8	51.4	107	53.0	58.9
Total	*502*	*100.0*	*100.0*	*202*	*100.0*	*100.0*

*Data Sources: National Cancer Registry Data, ONS LS

Table 2. Number and percentage of men and women with each cancer type who died within 1 and 5 years of their diagnosis compared to England & Wales, patients diagnosed 1 January 2008 and 30 April 2016.

Cancer	Men					Women				
	ONS-LS N	ONS-LS % 1y	E+W* % 1y	ONS-LS % 5y	E+W* % 5y	ONS-LS N	ONS-LS % 1y	E+W* % 1y	ONS-LS % 5y	E+W* % 5y
Breast	-	-	-	-	-	3473	5%	6%	18%	21%
Prostate	3044	7%	8%	23%	27%	-	-	-	-	-
Colorectal	1522	23%	25%	47%	51%	1237	24%	28%	46%	45%
NHL	483	23%	25%	39%	43%	372	20%	22%	36%	40%
Bladder	502	25%	28%	51%	55%	202	39%	41%	56%	59%

*Data Sources: National Cancer Registry Data, ONS-LS

Figure legends

Figure 1. Age-standardised net survival (ASNS) estimates (95% CI) for individual- compared to area-level measures of **education**: patients diagnosed 2008-2016

a) Men
b) Women

Figure 2. Age-standardised net survival (ASNS) estimates (95% CI) for individual- compared to area-level measures of **income**: patients diagnosed 2008-2016

a) Men
b) Women

Figure 3. Age-standardised net survival (ASNS) estimates (95% CI) for individual- compared to area-level measures of **occupation**: patients diagnosed 2008-2016

a) Men
b) Women

Figure 4. Comparison of individual vs. area-level deprivation gaps[a]

Footnote to Figure 4:

[a]Deprivation gaps are negative where survival is lower in the more deprived groups. The dashed line indicates where the gap according to individual socio-economic group and the area-level index is equal (i.e. y=x).

Footnote required for all Figures 1-4

Data source: ONS LS

For peer review only

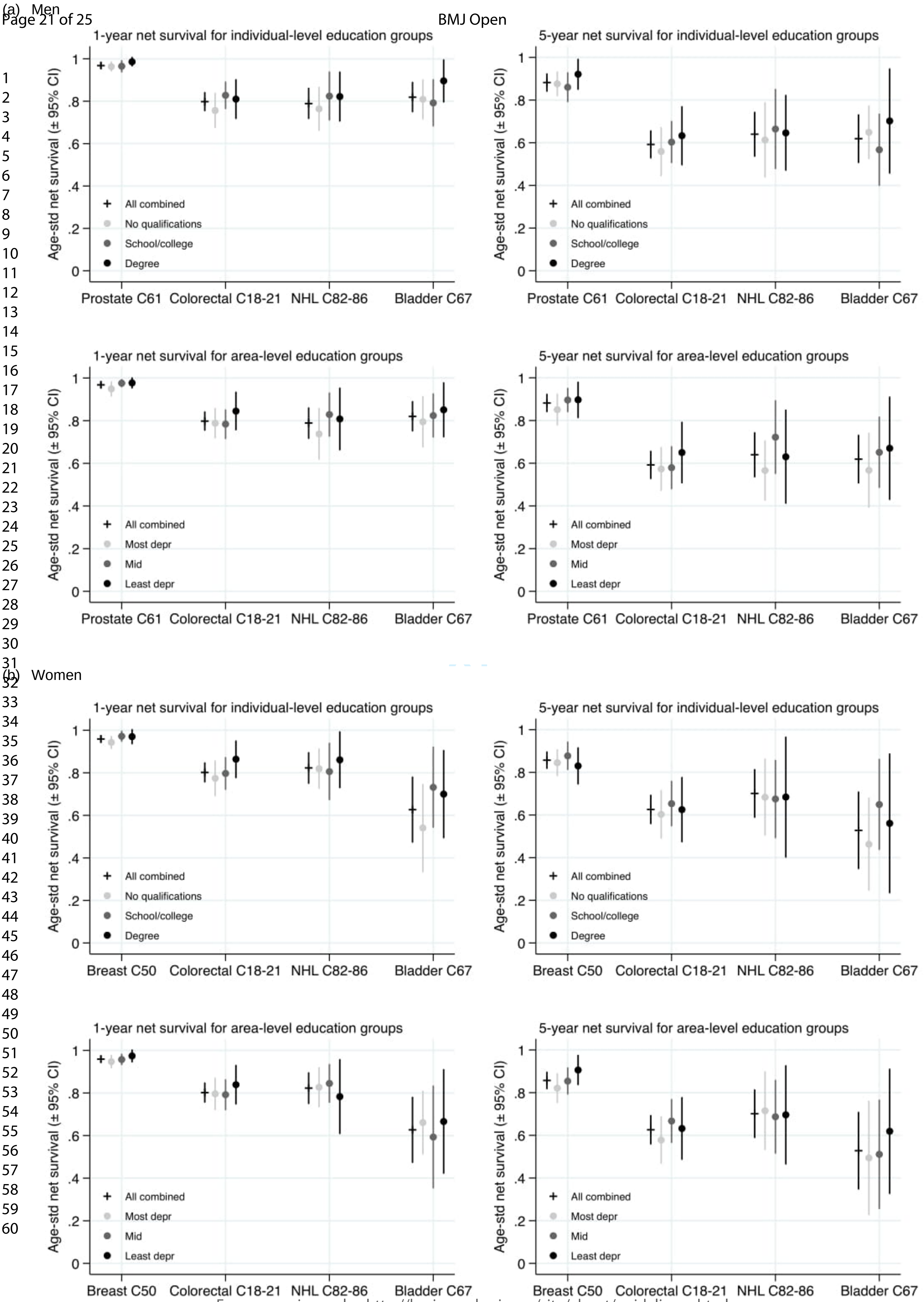
(a) Men
1-year net survival for individual-level education groups
5-year net survival for individual-level education groups
1-year net survival for area-level education groups
5-year net survival for area-level education groups
Age-std net survival (± 95% CI)
All combined
No qualifications
School/college
Degree
Most depr
Mid
Least depr
Prostate C61
Colorectal C18-21
NHL C82-86
Bladder C67
(b) Women
1-year net survival for individual-level education groups
5-year net survival for individual-level education groups
1-year net survival for area-level education groups
5-year net survival for area-level education groups
Breast C50
Colorectal C18-21
NHL C82-86
Bladder C67

(a) Men

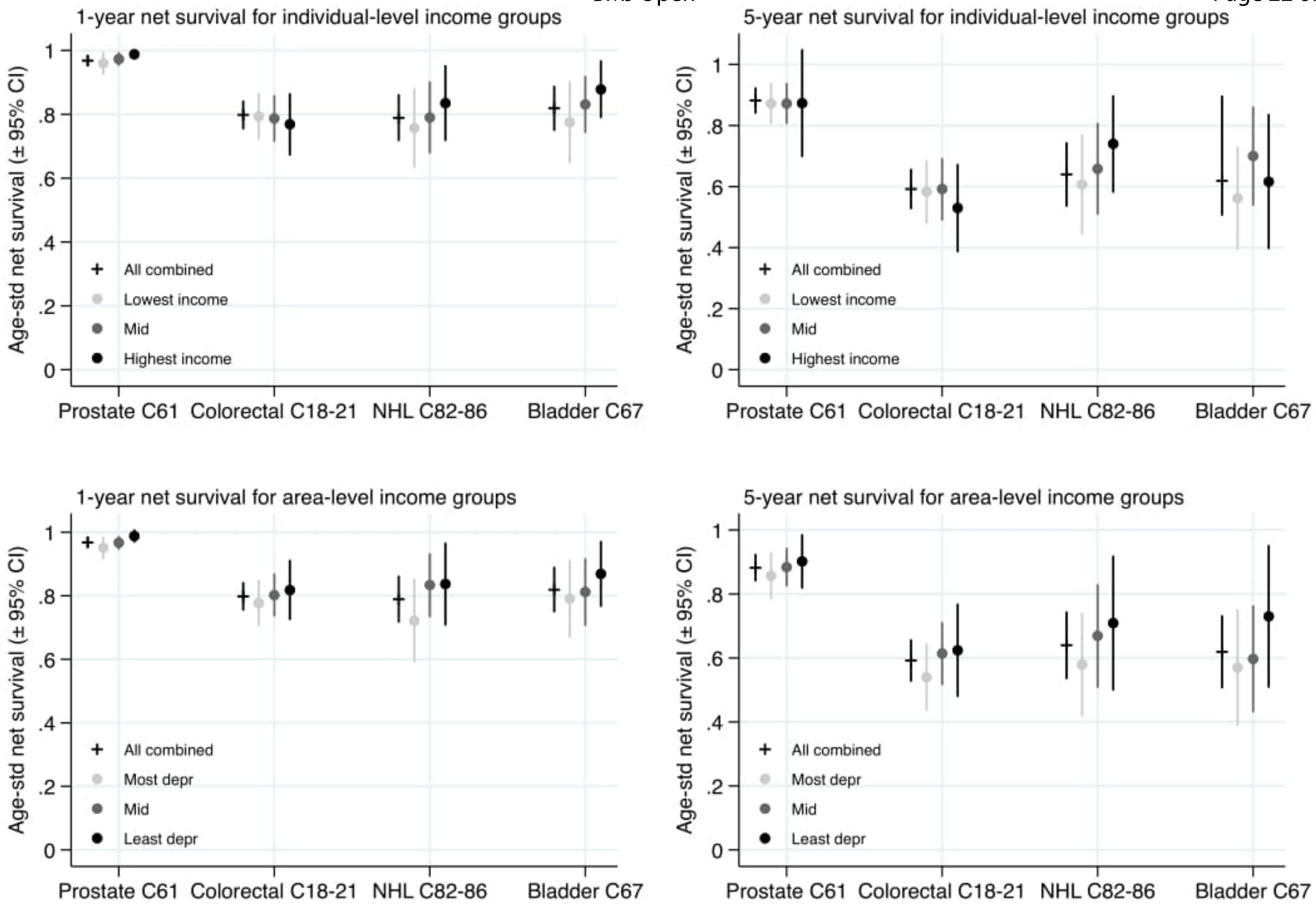
1-year net survival for individual-level income groups
5-year net survival for individual-level income groups
1-year net survival for area-level income groups
5-year net survival for area-level income groups
Age-std net survival (± 95% CI)
1
.8
.6
.4
.2
0
All combined
Lowest income
Mid
Highest income
Most depr
Least depr
Prostate C61
Colorectal C18-21
NHL C82-86
Bladder C67

(b) Women

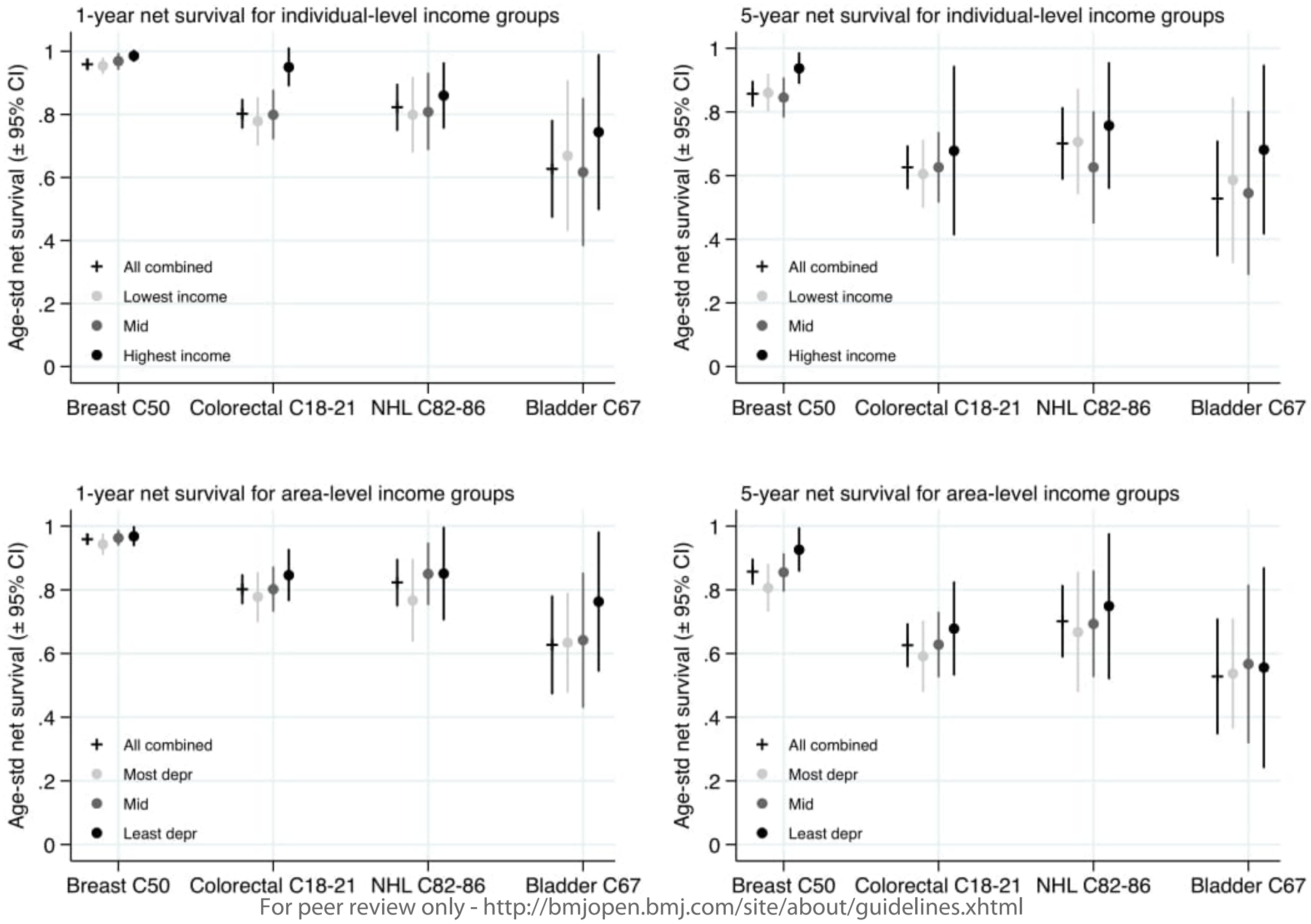
1-year net survival for individual-level income groups
5-year net survival for individual-level income groups
1-year net survival for area-level income groups
5-year net survival for area-level income groups
Age-std net survival (± 95% CI)
1
.8
.6
.4
.2
0
All combined
Lowest income
Mid
Highest income
Most depr
Least depr
Breast C50
Colorectal C18-21
NHL C82-86
Bladder C67

(a) Men

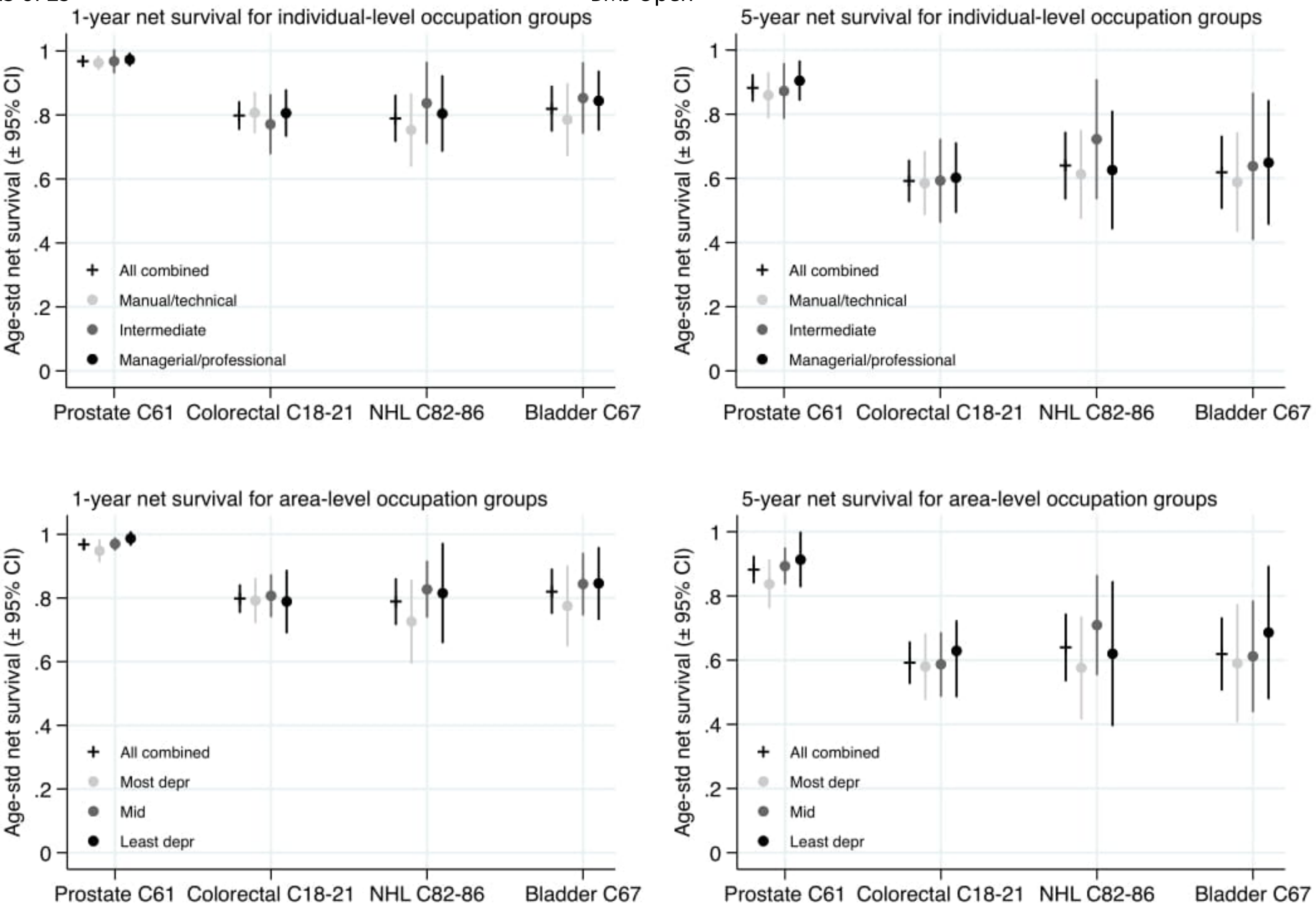
1-year net survival for individual-level occupation groups
5-year net survival for individual-level occupation groups
1-year net survival for area-level occupation groups
5-year net survival for area-level occupation groups
Age-std net survival (± 95% CI)
All combined
Manual/technical
Intermediate
Managerial/professional
Most depr
Mid
Least depr
Prostate C61
Colorectal C18-21
NHL C82-86
Bladder C67

(b) Women

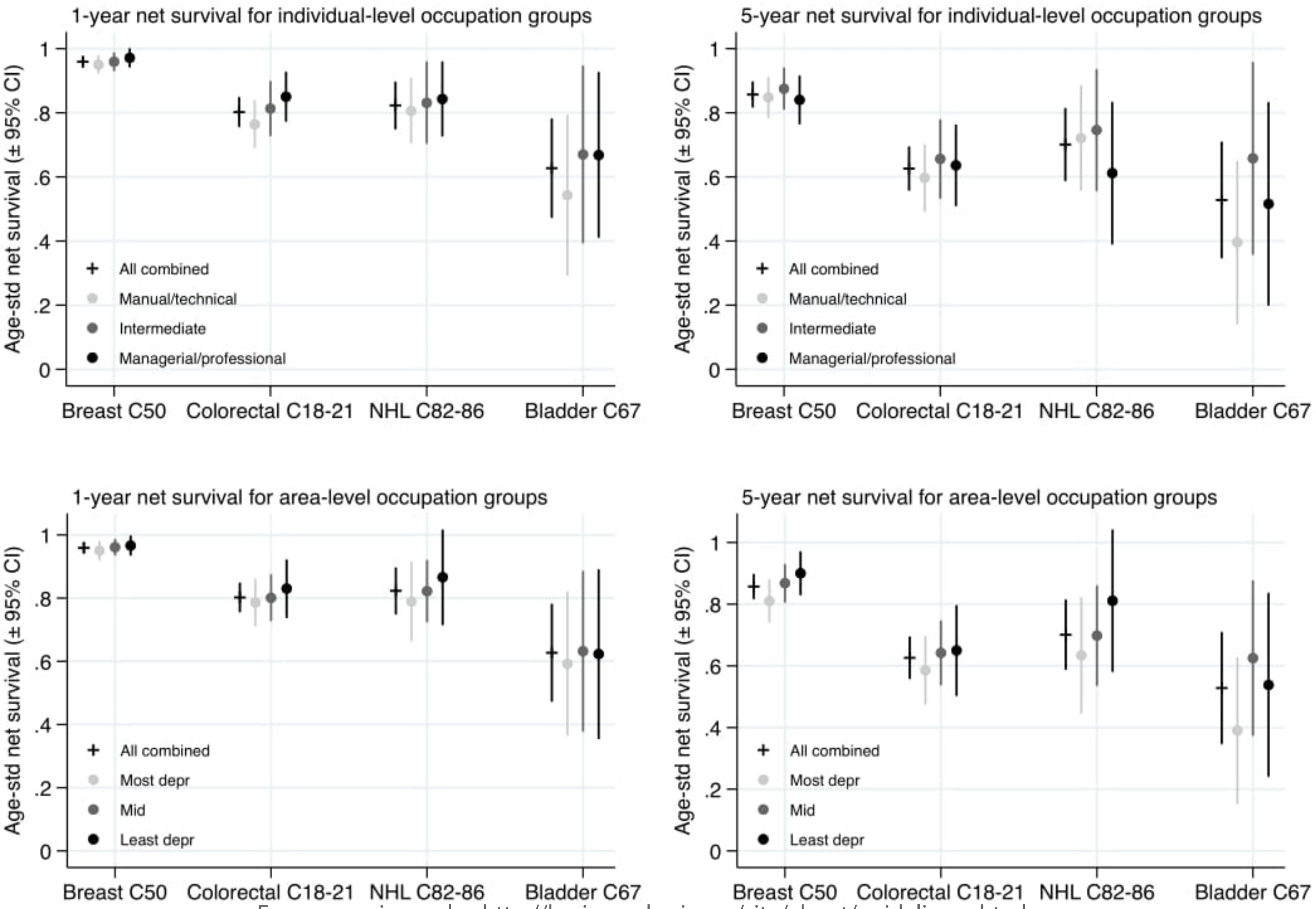
1-year net survival for individual-level occupation groups
5-year net survival for individual-level occupation groups
1-year net survival for area-level occupation groups
5-year net survival for area-level occupation groups
Age-std net survival (± 95% CI)
All combined
Manual/technical
Intermediate
Managerial/professional
Most depr
Mid
Least depr
Breast C50
Colorectal C18-21
NHL C82-86
Bladder C67

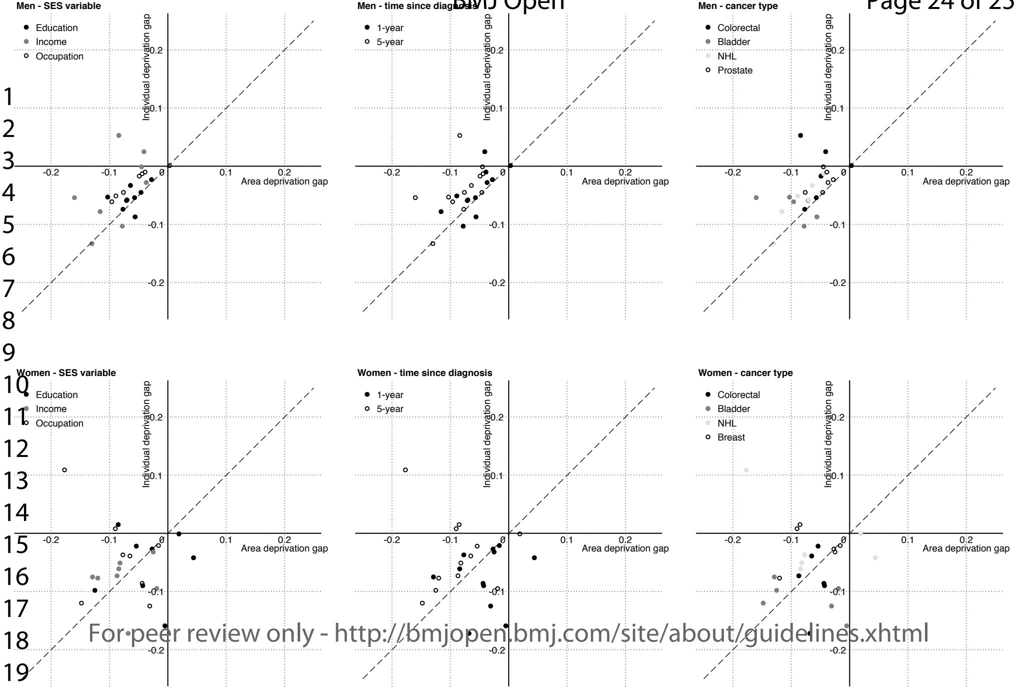

For peer review only - http://bmjopen.bmj.com/site/about/guidelines.xhtml

STROBE Statement—Checklist of items that should be included in reports of ***cohort studies***

	Item No	Recommendation	Page No
**Title and abstract**	1	(*a*) Indicate the study's design with a commonly used term in the title or the abstract	2
		(*b*) Provide in the abstract an informative and balanced summary of what was done and what was found	3
**Introduction**			
Background/rationale	2	Explain the scientific background and rationale for the investigation being reported	5
Objectives	3	State specific objectives, including any prespecified hypotheses	6
**Methods**			
Study design	4	Present key elements of study design early in the paper	6-7
Setting	5	Describe the setting, locations, and relevant dates, including periods of recruitment, exposure, follow-up, and data collection	6
Participants	6	(*a*) Give the eligibility criteria, and the sources and methods of selection of participants. Describe methods of follow-up	6, 7
		(*b*) For matched studies, give matching criteria and number of exposed and unexposed	N/A
Variables	7	Clearly define all outcomes, exposures, predictors, potential confounders, and effect modifiers. Give diagnostic criteria, if applicable	6, 7
Data sources/ measurement	8*	For each variable of interest, give sources of data and details of methods of assessment (measurement). Describe comparability of assessment methods if there is more than one group	6, 7
Bias	9	Describe any efforts to address potential sources of bias	
Study size	10	Explain how the study size was arrived at	6
Quantitative variables	11	Explain how quantitative variables were handled in the analyses. If applicable, describe which groupings were chosen and why	6, 7, 8
Statistical methods	12	(*a*) Describe all statistical methods, including those used to control for confounding	7, 8
		(*b*) Describe any methods used to examine subgroups and interactions	
		(*c*) Explain how missing data were addressed	
		(*d*) If applicable, explain how loss to follow-up was addressed	
		(*e*) Describe any sensitivity analyses	
**Results**			
Participants	13*	(a) Report numbers of individuals at each stage of study—eg numbers potentially eligible, examined for eligibility, confirmed eligible, included in the study, completing follow-up, and analysed	T1 T2
		(b) Give reasons for non-participation at each stage	
		(c) Consider use of a flow diagram	
Descriptive data	14*	(a) Give characteristics of study participants (eg demographic, clinical, social) and information on exposures and potential confounders	8, T1, T2
		(b) Indicate number of participants with missing data for each variable of interest	T1
		(c) Summarise follow-up time (eg, average and total amount)	T1
Outcome data	15*	Report numbers of outcome events or summary measures over time	T1, 8, 9

For peer review only - http://bmjopen.bmj.com/site/about/guidelines.xhtml

Main results	16	(*a*) Give unadjusted estimates and, if applicable, confounder-adjusted estimates and their precision (eg, 95% confidence interval). Make clear which confounders were adjusted for and why they were included<br>(*b*) Report category boundaries when continuous variables were categorized<br>(*c*) If relevant, consider translating estimates of relative risk into absolute risk for a meaningful time period	8, 9
Other analyses	17	Report other analyses done—eg analyses of subgroups and interactions, and sensitivity analyses	
**Discussion**			
Key results	18	Summarise key results with reference to study objectives	9, 10
Limitations	19	Discuss limitations of the study, taking into account sources of potential bias or imprecision. Discuss both direction and magnitude of any potential bias	11
Interpretation	20	Give a cautious overall interpretation of results considering objectives, limitations, multiplicity of analyses, results from similar studies, and other relevant evidence	10,11
Generalisability	21	Discuss the generalisability (external validity) of the study results	11, 12
**Other information**			
Funding	22	Give the source of funding and the role of the funders for the present study and, if applicable, for the original study on which the present article is based	14

*Give information separately for exposed and unexposed groups.

Note: An Explanation and Elaboration article discusses each checklist item and gives methodological background and published examples of transparent reporting. The STROBE checklist is best used in conjunction with this article (freely available on the Web sites of PLoS Medicine at http://www.plosmedicine.org/, Annals of Internal Medicine at http://www.annals.org/, and Epidemiology at http://www.epidem.com/). Information on the STROBE Initiative is available at http://www.strobe-statement.org.

For peer review only
